# Instructed and Acquired Contingencies in Response-Inhibition Tasks

**DOI:** 10.5334/joc.53

**Published:** 2019-02-04

**Authors:** Maisy Best, Ian P. L. McLaren, Frederick Verbruggen

**Affiliations:** 1 University of Glasgow, UK; 2 University of Exeter, UK; 3 Ghent University, BE

**Keywords:** Cognitive control, Executive functions, Learning

## Abstract

Inhibitory control can be triggered directly via the retrieval of previously acquired stimulus-stop associations from memory. However, a recent study suggests that this item-specific stop learning may be mediated via expectancies of the contingencies in play (Best, Lawrence, Logan, McLaren, & Verbruggen, 2016). This could indicate that stimulus-stop learning also induces strategic proactive changes in performance. We further tested this hypothesis in the present study. In addition to measuring expectancies following task completion, we introduced a between-subjects expectancy manipulation in which one group of participants were informed about the stimulus-stop contingencies and another group did not receive any information about the stimulus-stop contingencies. Moreover, we combined this instruction manipulation with a distractor manipulation that was previously used to examine strategic proactive adjustments. We found that the stop-associated items slowed responding in both conditions. Furthermore, participants in both conditions generated expectancies following task completion that were consistent with the stimulus-stop contingencies. The distractor manipulation was ineffective. However, we found differences in the relationship between the expectancy ratings and task performance: in the instructed condition, the expectancies reliably correlated with the response slowing for the stop-associated items, whereas in the uninstructed condition we found no reliable correlation. These differences between the correlations were reliable, and our conclusions were further supported by Bayesian analyses. We conclude that stimulus-stop associations that are acquired either via task instructions or via task practice have similar effects on behaviour but could differ in how they elicit response slowing.

‘Response inhibition’ is often considered to be one of the core executive control functions, and the concept has been linked to flexible and adaptive behaviour in healthy and clinical populations (e.g. Chambers, Garavan, & Bellgrove, 2009; Miyake et al., 2000; Ridderinkhof, van den Wildenberg, Segalowitz, & Carter, 2004; Verbruggen & Logan, 2008a). However, our previous work suggests that stopping in go/no-go and stop-signal tasks can be ‘automatically’ triggered by the retrieval of instances or episodes from memory (Verbruggen & Logan, 2008b; for a review, see Verbruggen, Best, Bowditch, Stevens, & McLaren, 2014), without much involvement of ‘top-down’ control mechanisms.

In the go/no-go paradigm, participants respond when a go stimulus is presented and withhold their response when a no-go stimulus is presented. In the stop-signal paradigm, participants usually perform a choice reaction-time task on no-signal trials; on a random selection of trials, a stop signal (e.g. an auditory tone or an extra visual cue) is presented after a variable delay (stop-signal delay; SSD) which instructs participants to withhold their response to the go stimulus. Several studies have manipulated the consistency of the mapping between specific stimuli and stopping in these response-inhibition tasks. These studies have demonstrated that, following a training phase, responding is typically slower for old stop-associated stimuli compared with consistent go stimuli (always presented on no-signal trials), inconsistent stimuli (presented on no-signal and stop-signal trials with equal probability), or new stimuli (not previously presented; e.g. Best, Lawrence, Logan, McLaren, Verbruggen, 2016; Bowditch, Verbruggen & McLaren, 2016; Chiu, Aron, & Verbruggen, 2012; Liefooghe, Degryse, Theeuwes, 2016; Verbruggen, Best, et al., 2014; Verbruggen & Logan, 2008b). At least in some paradigms, this slowing can be attributed to the retrieval of associations between specific stimuli and the stop ‘goal’ or ‘response’, such that the stimulus (e.g. ‘apple’) or stimulus category (e.g. ‘fruit’) becomes associated with stopping (cf. Logan, 1988). When the stimulus (or category) is repeated, the stimulus-stop association is retrieved, interfering with responding on no-signal trials. Thus, response inhibition is not necessarily an entirely instruction-based (e.g. ‘stop when an extra signal occurs’) act of executive control, it can also be triggered by the direct retrieval of a ‘stop response’ from memory (even when it is not required or intended at a given moment).

The ‘automatic^1^ inhibition’ account states that after stimulus-specific stop training the need for rule- or instruction-based control is reduced and may eventually disappear altogether as control is triggered ‘bottom-up’ following stimulus presentation (see also e.g. Chein & Schneider, 2012). In some situations, however, slowing for old stop words could also be caused by anticipatory processes. One of the main functions of the control system is biasing competition between stimulus or response options on the basis of expectancies or rules. When a certain action is predicted, for example, the motor network is pre-activated, biasing action selection and reducing the latency of the anticipated action (e.g. Bestmann, 2012; Meyer & Kieras, 1997; but see also Verbruggen, McAndrew, Weidemann, Stevens, & McLaren, 2016). Consistent with this idea, we recently found that response slowing for old stop-associated items may have been mediated via expectancies of the stimulus-stop contingencies in play (Best et al., 2016; Best & Verbruggen, under review). Following task completion, participants were required to rate how much they expected to withhold their response to each of the stimuli presented in the task. We found correlations between the expectancy ratings and response slowing in some of these experiments. This suggests that expectancies generated on the basis of the acquired stimulus-stop mappings may contribute to response slowing. After all, previous work shows that when instructions at the beginning of a block or a cue at the beginning of a trial indicate that a no-go or stop signal may appear, participants tend to proactively adjust attentional settings, increase response thresholds, or proactively suppress motor output to enhance detection of no-go or stop signals and prevent premature go responses (e.g. Aron, 2011; Elchlepp, Lavric, Chambers, & Verbruggen, 2016; Jahfari, Stinear, Claffey, Verbruggen, & Aron, 2010; Verbruggen & Logan, 2009a; Verbruggen, Stevens, & Chambers, 2014; Zandbelt, Bloemendaal, Neggers, Kahn, & Vink, 2012). Our expectancy results (Best et al., 2016) suggest that stop-associated items could become such stop cues (e.g. ‘if stimulus X then *p*(stop) is high’). In this case, the slowing observed for old stop-associated items could be due to deliberate and strategic proactive control adjustments of attentional, response-selection, or motor settings on go trials rather than suppression of motor output via the direct retrieval of stimulus-stop associations (i.e. stimulus X = stop).

Evidence for the involvement of strategic proactive control adjustments in stop-training tasks would have two important implications. First, it would contradict the idea that inhibitory control is always fully ‘automatised’ after practice in stop-training tasks. Second, it would have important practical implications as it could shed a new light on findings in the applied inhibition training domain. Studies of this type suggest that the acquisition of stimulus-stop associations could be an effective way to reduce engagement in impulsive and compulsive behaviours, such as excessive unhealthy food intake (e.g., Adams, Lawrence, Verbruggen, & Chambers, 2017; Houben, 2011; Houben & Jansen, 2011; Lawrence et al., 2015a, 2015b; Veling, Aarts, & Papies, 2011; Veling, Aarts, & Stroebe, 2012) and alcohol consumption (e.g., Houben, Nederkoorn, Wiers & Jansen, 2011; Jones & Field, 2013; for meta-analyses, see Allom, Mullan, & Hagger, 2015; Jones et al., 2016). Our previous expectancy findings (Best et al., 2016) indicate that the effects of inhibition training could be more strategic than initially thought.

The ‘direct activation’ (i.e. the stop process is directly activated by the retrieval of stimulus-stop associations) and ‘strategic proactive adjustment’ (i.e. go processing is proactively adjusted when the probability of a stop signal is deemed high) accounts both predict increased reaction times (RTs) for old stop-associated items. One way to assess whether participants proactively adjust response strategies for old stop-associated items is to question them at the end of the experiment (as in Best et al., 2016). However, measuring knowledge of rules, expectancies, or strategies at the end of the experiment has limitations (Newell & Shanks, 2014; Shanks, 2010). Newell and Shanks (2014) proposed four criteria for the assessment of awareness: (1) *reliability* (the assessment should not be affected by factors that did not also affect behavioural performance); (2) *relevance* (assessments should only target information relevant to the behaviour in question); (3) *sensitivity* (assessments should be made under optimal conditions, such as using the same cues as in the task); and (4) *immediacy* (the assessment should be made during behaviour or immediately afterwards). Whilst assessing expectancies at the end of task performance might meet the first three criteria, it is certainly sub-optimal that expectancies were obtained after learning had taken place. However, asking participants to provide an expectancy rating at the beginning of each trial (as in Best et al., 2016, Experiment 4) could change the nature of the task, and may ‘push’ participants towards strategic proactive adjustments in anticipation of a stop signal. Therefore, in the present study, we used a complementary approach to assess the contribution of strategic proactive adjustments that did not require participants to provide a trial-by-trial expectancy rating. In addition to measuring expectancy ratings at the end of the experiment, we used a distractor manipulation to further determine how slowing to stop-associated items is achieved.

Verbruggen, Stevens, and Chambers (2014) introduced perceptual (visual) distractors to measure reactive and (strategic) proactive control during performance of a stop-signal task. In their version of the task, there were three block types: central-signal blocks (a visual stop signal could occur in the centre of the screen), non-central signal blocks (a visual stop signal could occur in the periphery), and no-signal blocks (no stop signals could occur). Moreover, they presented random two-letter strings as visual distractors on a random 50% of trials. This created a trade-off in non-central blocks: strategic proactive adjustments involved the widening of attentional focus to detect stop signals in the periphery whereas interference control required the narrowing of focus to avoid processing the distractors. These opposing demands produced a larger distractor effect^2^ on no-signal trials in non-central signal blocks than on no-signal trials in the two other block types.

In the present study, we used the same non-central signals (i.e. visual stop signals that occurred in the periphery of the screen) and distractor manipulation (random two-letter strings) to examine if strategic proactive control adjustments were made for stop-associated items. We reasoned that if stop-associated items become cues for strategic proactive control adjustments, participants would proactively widen their attention in order to detect the stop signal in the periphery. As shown in Verbruggen, Stevens et al. (2014), widening of attention would, in turn, result in greater processing of the distractors and thus increase the size of the distractor effect (in analogy with the block-based differences observed in Verbruggen, Stevens, et al., 2014). In contrast, the ‘direct activation’ account does not predict a widening of attention as the stop-associated items would directly activate the stop goal and so participants would not need to rely on the stop signal as a cue to stop their response. Thus, according to the ‘direct activation’ account, there would be no widening of attention and so no change in the size of the distractor effect between items consistently associated with stopping and items consistently associated with going or associated with both going and stopping.

A pilot study (reported in Supplementary Materials) showed that words frequently associated with stopping did not display a larger distractor effect than words rarely or never associated with stopping. As noted in the previous paragraph, the ‘strategic proactive control adjustment’ account predicts that items frequently associated with stopping should lead to a more pronounced widening of attention (as reflected by an increased distractor effect) compared with items never or rarely associated with stopping. There was no evidence of this data pattern in the pilot study. Thus, these findings argue against the ‘strategic proactive control adjustment’ account. However, on the basis of the pilot study, it is not possible to determine whether participants did not make strategic proactive adjustments to their attention or whether the distractor manipulation did not detect such adjustments. In the main study, we therefore decided to combine the distractor manipulation (used in the pilot study) with a between-subjects instruction manipulation that was designed to increase strategic proactive control adjustments in one group of participants. In the ‘instructed’ group, participants were shown a list of the words frequently associated with stopping at the beginning of each block and were explicitly informed that these words were more likely to occur with a stop signal than other words in the task. The reasoning was that, if any strategic proactive control adjustments occur at all, they should be more pronounced in the instructed group (as measured by a larger distractor effect for these ‘stop-associated’ words) compared with another group that did not receive any information about the stop-associated words and so acquired these associations through task practice alone (as in the pilot study). In other words we expected that the stop-associated words in the instructed condition would act like instructed cues (as in other studies that examined strategic proactive adjustments; e.g. Aron, 2011; Elchlepp, Lavric, Chambers, & Verbruggen, 2016; Jahfari, Stinear, Claffey, Verbruggen, & Aron, 2010; Verbruggen & Logan, 2009a; Zandbelt, Bloemendaal, Neggers, Kahn, & Vink, 2012). Thus, by contrasting performance in the instructed group with performance in an uninstructed group (in which participants were not told about the stimulus-stop contingencies) we could test how the acquisition of instructed and uninstructed stimulus-stop contingencies influenced performance in a stop-signal task.

Furthermore, in addition to the instruction manipulation, we also obtained expectancy ratings at the end of the experiment (unlike in the pilot study). By comparing the correlation between these ratings and performance in both conditions, we also hoped to find out more about how instructions and learning based on practice would influence performance.

## The present study

In the present study, we manipulated task instructions and perceptual distractors to investigate how expectancies influence learning in a stop-signal task and so distinguish the contributions of strategic proactive adjustments and the direct retrieval of the stop goal.

### The main design

**Figure 1:**
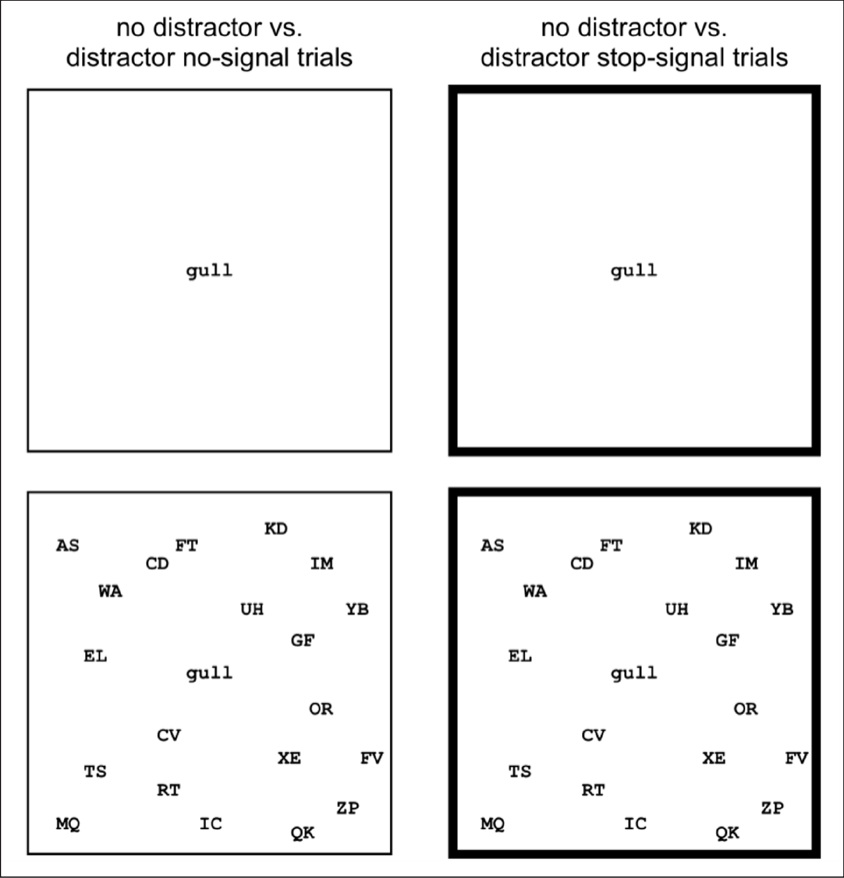
The distractor stop task. A word was presented in the middle of a square. Participants had to judge whether the word referred to a natural or human-made object. On half of the trials (distractor trials), randomly generated two-letter strings appeared at random locations every 100 ms. On some trials (stop-signal trials), the square turned bold after a variable delay from the onset of the word, instructing the participants to stop their response. For display purposes, foreground and background colours are switched in this figure. In the present study, white stimuli appeared against a black background.

On each trial, a large square and a fixation signal were presented in the middle of the screen (Figure 1). After a delay, a single word appeared in the centre of the square. Participants had to decide whether the word referred to a natural or a human-made object. On stop-signal trials, the lines of the surrounding square became thicker (i.e. the stop signal), instructing the participants to stop their response. There were three word types in this experiment: *80%-stop words* (on 80% of trials where this particular word type was presented, a stop signal was presented); *20%-stop words* (on 20% of trials where this particular word type was presented, a stop signal was presented); and *0%-stop words* (only occurred on no-signal trials). The 80%-stop words, 20%-stop words, and 0%-stop words remained the same throughout the experiment.

To contrast the ‘direct activation’ and ‘strategic proactive adjustment’ accounts, we presented visual distractors to determine if slowing was (at least partly) due to strategic proactive changes in attentional settings. In addition, we directly manipulated contingency knowledge via task instructions (between-subjects). In the instructed condition, participants were presented with a list of the 80%-stop words at the beginning of each block; in the uninstructed condition, participants received no information about the stimulus-stop contingencies. In the instructed condition, participants received no information about the 20%-stop and 0%-stop words, although it is possible that our instruction manipulation might have influenced more general knowledge about the existence of associations within the task (Gaschler, Marewski, Wenke, & Frensch, 2014). We return to this issue in the General Discussion.

We also examined how performance changed across the task. Instruction following is implemented via a fast prefrontal (indirect) route that links stimuli and responses whereas practice-based learning would be implemented via a slower (direct) route that includes stimulus areas, response areas, and the basal ganglia (e.g. Chein & Schneider, 2012; Ramamoorthy & Verguts, 2012). Therefore we examined how performance changed over three experiment parts.

### The predictions

We used a ‘triangulation’ approach to distinguish between the ‘direct activation’ and ‘strategic proactive adjustment’ accounts.

First, we predicted that responding on no-signal trials would be slower and the probability of responding on stop-signal trials would be lower for the 80%-stop words compared with the other the word types in both the instructed and uninstructed conditions. Furthermore, if the 80%-stop words elicited extreme slowing or outright stopping we would detect this as a higher probability of missed responses on no-signal trials for the 80%-stop words than for the other word types. We further predicted that the slowing for the 80%-stop words would be especially pronounced in the instructed condition (as reflected in a reliable two-way interaction between word type and condition). More pronounced slowing for the 80%-stop words in the instructed than in the non-instructed condition would provide a critical manipulation check that participants in the instructed condition used the instructed expectancies during task performance.

Second, stimulus-stop learning does not seem to modulate attentional processes much when visuospatial stop signals are used (Best & Verbruggen, under review) whereas proactive control adjustments do seem to modulate attentional processes (Verbruggen, Stevens, et al., 2014; see also e.g. Elchlepp et al., 2016; Schevernels et al., 2015). Thus, we predicted that the distractor effect (as measured in RTs) would be larger for the 80%-stop words than for the other word types if participants made strategic proactive adjustments (similar to the non-central vs. control blocks in Verbruggen, Stevens, et al., 2014). As strategic proactive adjustments seemed more likely in the instructed condition than in the uninstructed condition, we also predicted a reliable three-way interaction between word type, condition, and distractor. Importantly, the absence of a reliable three-way interaction in combination with the presence of a reliable two-way interaction between distractor and word type would indicate that strategic proactive adjustments were made in both instruction conditions.

Third, we hypothesised that the slowing (or even outright stopping, as reflected in increased probability of missed responses on no-signal trials and decreased probability of responding on stop-signal trials) to the stop-associated words in the uninstructed condition would emerge with task practice. This would reflect the acquisition of the stimulus-stop associations through learning. In contrast, as participants in the instructed condition were shown a list of the words frequently associated with stopping at the beginning of each block, we predicted that slowing or outright stopping to the stop-associated items in the instructed condition would be present across task practice (but could get stronger across task practice if additional learning took place). Thus, we analysed how task performance changed across three parts. Importantly, if the difference between the instructed and uninstructed groups is only due to the rate of learning (as discussed above), our effects should also be modulated by part. More specifically, the effect of word type would differ in the two conditions at the beginning of the task but not towards the end of the task.

Finally, at the end of the experiment, we obtained an expectancy rating for each of the words presented in the task. We predicted that participants would expect to stop more to the 80%-stop words than the other word types following task completion, especially in the instructed condition. This would reflect the acquisition of the stimulus-stop expectancies instructed at the beginning of each block in the instructed condition. We further hypothesised that the expectancy ratings would correlate with the slowing for the 80%-stop words during task performance in the instructed condition, showing the slowing to be driven (in part) by strategic adjustments to task performance on the basis of the acquisition or formation of expectancies. Thus, a correlation between slowing for the 80%-stop words and expectancy would provide evidence for the ‘strategic proactive adjustment’ account. Since the ‘direct activation’ account predicts that slowing for the 80%-stop words would be driven by direct activation of the stop goal from memory, we hypothesised that there would be no correlation between the expectancy ratings and the slowing for the 80%-stop words in the uninstructed condition. However, if the 80%-stop words acted as similar stop expectancy ‘cues’, we should see a similar positive correlation in the uninstructed condition.

## Experiments

There were two experiments. In Experiment 1, we encouraged fast responding using a strict response deadline (1250 ms). We also obtained eye-movement data as an additional dependent variable to detect within-trial shifts of attentional focus. However, the probability of missed no-signal responses (*M* = 0.050, *SD* = 0.066) was higher than in our previous study (Best et al., 2016), including for items that were not associated with stopping. This indicates that the response deadline was too strict. Therefore, we ran another experiment in which we extended the response deadline (2000 ms) to reduce the probability of missed responses. To ensure that the overall task duration was comparable across experiments, we did not obtain eye-movement data in Experiment 2. After all, the eye-movement data of Experiment 1 did not substantially add to the information provided by the behavioural data (Supplementary Materials; this is consistent with Best & Verbruggen, under review; Verbruggen, Stevens, et al., 2014). Note that the overall study set-up remained the same.

Initial analyses revealed that whilst the probability of missed no-signal responses was reduced in Experiment 2 (and the no-signal reaction times numerically increased) the overall pattern of results (i.e. interactions between word types, distractor types, and instruction conditions) was consistent across both experiments. Therefore, we combined the data of both experiments as this increased the overall power (for completeness, we present the separate analyses for the experiments in the Supplementary Materials).

### Method

**Participants.** 120 volunteers (48 in Experiment 1 and 72 in Experiment 2) from the University of Exeter participated for monetary compensation (£8) or partial course credit (*M* = 19.88 years, *SD* = 3.05, 98 females, 109 right-handed). Two participants were replaced because the percentage of correct no-signal trials was below 70% (this exclusion criterion was based on our earlier pilot study; for details, see Supplementary Materials) and three participants in Experiment 1 were replaced due to poor calibration of the eye-tracker in one or more task blocks.

The experiments were approved by the local research ethics committee (School of Psychology, University of Exeter). Each participant provided informed consent after the nature and possible consequences of the study was explained. The target sample size and exclusion criteria were decided in advance of data collection for each experiment to ensure that we had enough power (0.80) to detect small-to-medium sized effects (*f* = 0.20) in the word type by condition interactions. A power calculation was performed using G*Power 3.1 (Faul, Erdfelder, Lang, & Buchner, 2009). Participants were randomly assigned to between-subjects groups.

**Apparatus and stimuli.** The experiments were run using Psychtoolbox (Brainard, 1997). The stimuli were presented on a 17-in CRT monitor (screen size: 1024 × 768 pixels) in Experiment 1 and on a 21-in iMac (screen size: 1920 × 1080) in Experiment 2. In Experiment 1, an EyeLink 1000 Desktop Mount camera system (SR Research, Ottawa, Canada), calibrated before each block, tracked the gaze position of the right eye during the whole block.

The stimuli consisted of a large square (350 × 350 pixels) and a word in white lowercase font (Courier 16 point) on a black background (Figure 1). The stimuli were presented in the centre of the screen. We created a list of 50 four-letter words (see Supplementary Materials), which could refer to natural or human-made objects. The experiment consisted of three parts, with five blocks per part. Each word was presented twice per block (1x with and 1x without distractors). There were three different word types: *80%-stop words* (10 words; on 80% of the trials, a stop signal was presented); *20%-stop words* (30 words; on 20% of the trials, a stop signal was presented); and *0%-stop words* (10 words; these words could only occur on no-signal trials). There were more 20%-stop words than 80%-stop words to keep the overall probability of stop-signal trials low (0.28; see also Verbruggen & Logan, 2008b). Words were counterbalanced over conditions between participants.

**Procedure.** On each trial, the square and a fixation signal were presented in the middle of the screen. After 250 ms, the word replaced the fixation signal in the centre of the square. Half of the participants pressed the ‘c’ key (with their left index finger) when the word referred to a natural object, and the ‘m’ key (with their right index finger) when the word referred to a human-made object. This mapping was reversed for the other participants. On 50% of the trials, twenty two-letter randomly generated uppercase strings appeared in random locations within the square (distractor trials; Figure 1). To avoid overlap between these distractors and the word, the centre of the distractors was outside a smaller central region (100 × 50 pixels). New distractors were presented in different locations every 100ms throughout the duration of the trial. Participants had to ignore the distractors.

On stop-signal trials, the outer square turned bold (1 to 3 pixels) after a variable SSD, instructing participants to withhold their response. The stop signal occurred equally often on distractor and on non-distractor trials. The SSD for 20%-stop words was initially set at 500 ms and was continuously adjusted to obtain a probability of successful stopping of 0.50: the SSD decreased by 50 ms following an unsuccessful stop-signal trial, but increased by 50 ms following a successful stop. We used two separate one-up/one-down tracking procedures for distractor and no-distractor trials. The SSD for the 80%-stop words was yoked to the SSD for the corresponding 20%-stop words.

After the response deadline elapsed (1250 ms in Experiment 1; 2000 ms in Experiment 2) we presented feedback (on no-signal trials: ‘correct’, ‘incorrect’, or ‘not quick enough’ in case the participant did not respond before the end of the trial; on stop-signal trials: ‘correct stop’ or ‘failed stop’), which remained on the screen for 500 ms. The feedback was presented to encourage fast and accurate responding. The next trial started immediately after the feedback.

Stimulus-stop contingency knowledge was manipulated between-subjects. Participants in the instructed condition were presented with a 10-item list of the 80%-stop words on the screen at the beginning of each block and were instructed ‘*For certain words, the lines of the surrounding square will become thicker* (*indicating that you have to withhold your response*) *more often than for other words. These words are listed below*’. The stimulus-stop contingencies remained the same throughout the whole experiment so the same list was presented at the beginning of each block. No information about the 20%-stop and 0%-stop words was provided. At the beginning of each block, participants were instructed to remember as many of the words as possible. Once participants had done so, they pressed the ‘s’ key to move on to the next screen. There was no deadline on the word list screens. Participants in the uninstructed condition were not provided with any information about the stimulus-stop contingencies at the beginning of the blocks; the instruction screen simply said that they had to press the ‘s’ key to start the first trial. At the end of each block, we presented as feedback to the participants in both groups their mean RT on no-signal trials, the number of no-signal errors, the number of missed no-signal responses, and the percentage of failed stops.

Following completion of the experimental task, each word was again presented. The order of the words was randomised anew for each participant. Participants were asked to rate ‘how much do you expect to withhold your response when this word is presented?’ on a scale between 1 (‘*I definitely do not think this word indicates that I have to withhold my response*’) and 9 (‘*I definitely think this word indicates that I have to withhold my response*’). There was no response deadline for the expectancy ratings.

**Analyses.** All data processing and analyses were completed using R (R Development Core Team, 2014). All data files and R scripts are deposited in the Open Science Framework (https://osf.io/kydw6/?view_only=131cbcc73fce4abfbfc34375819ecaad).

To determine how learning and the presentation of the distractors influenced go performance, we analysed mean RT of correct no-signal trials. We also analysed the proportion of missed no-signal trials as a measure of outright stopping and/or extreme slowing (missed/(correct+incorrect+missed)). The proportion of incorrect trials ((incorrect/(correct+incorrect)); see Verbruggen & Logan, 2009b) was only analysed for completeness, as we did not have any strong predictions about how learning or the distractors could influence error rates (e.g. in learning did not affect error rates much in Verbruggen & Logan, 2008b; similarly, distractors did not influence error rates much in Verbruggen, Stevens, et al., 2014). To determine if learning influenced stopping performance, we analysed the probability of responding on stop-signal trials (*p*(respond|signal)) as the same SSD was used for 20%- and 80%-stop words (see also Best et al., 2016; Bowditch, Verbruggen, et al., 2016; Noël, Brevers, et al., 2016).

Performance was analysed with ANOVAs as a function of part (part: 1 = blocks 1–5; 2 = blocks 6–10; 3 = blocks 11–15), word type (80%-, 20%-, 0%-stop words), distractor type (distractor, no distractor), and instruction condition (instructed, uninstructed). As there were a high number of (possible) tests in the ANOVAs, we corrected the alpha level using the sequential Bonferroni procedure to correct for multiplicity (Cramer, et al., 2016). Where appropriate, we applied the Huyhn-Feldt correction for violations of sphericity. For pairwise comparisons, Hedge’s g_av_ is the reported effect size measure (Lakens, 2013).

For the no-signal RT analyses, we calculated Bayes factors for all main effects and interaction contrasts in the ANOVA design (Rouder, Morey, Speckman, & Province, 2012). We calculated these with the BayesFactor package in R, using the default prior (0.707; Morey, Rouder, & Jamil, 2015). We used a top-down approach to reduce the number of model comparisons. Top-down model comparisons investigate the effect of removing each fixed factor and interaction from the overall model, such that the removal of meaningful factors or interactions will have a deleterious effect on the model fit whereas the removal of non-meaningful factors or interactions will not.

**Figure 2:**
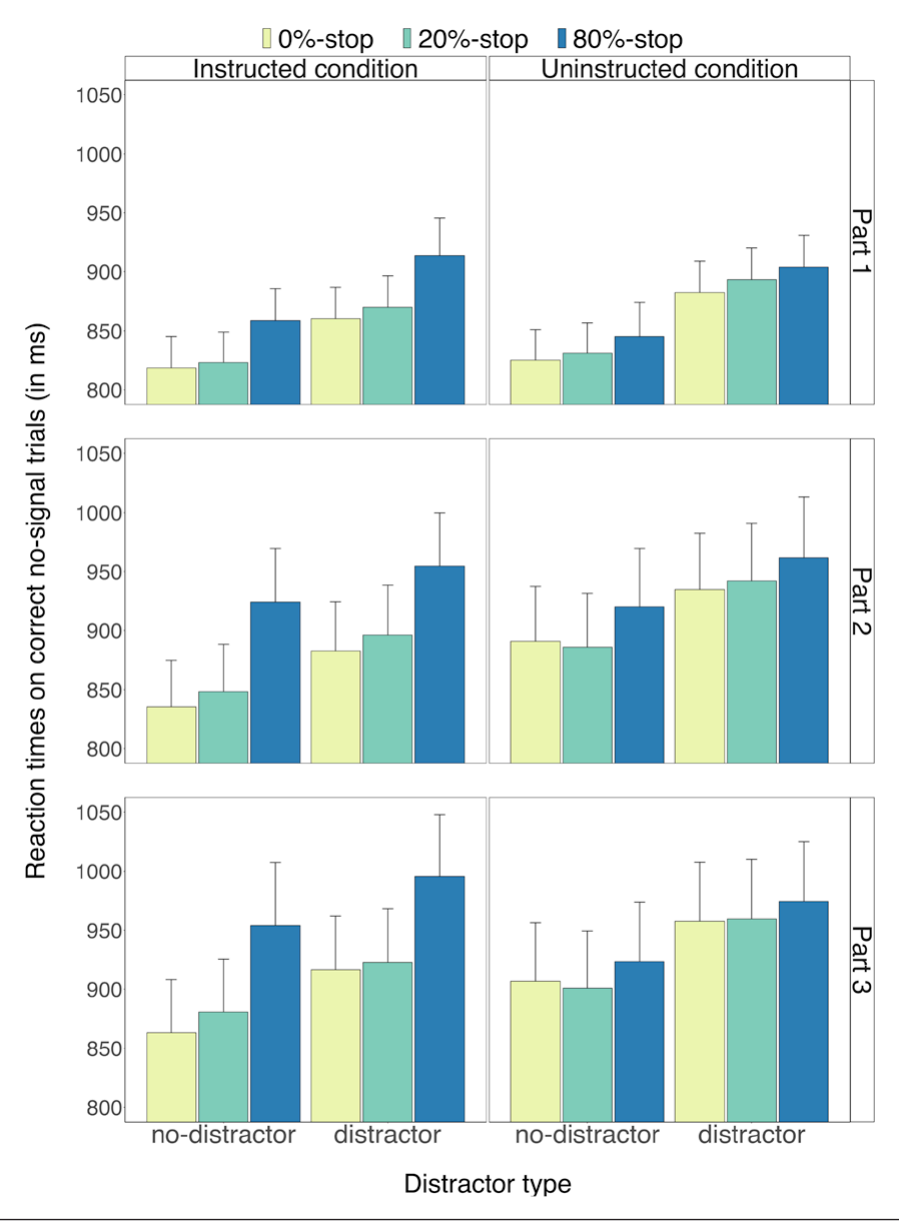
Reaction times on correct no-signal trials for the instructed condition (left panels) and for the uninstructed condition (right panels) as a function of part (1–3), word type (0%-stop, 20%-stop or 80%-stop words) and distractor type (distractor, no-distractor). Error bars are 95% confidence intervals.

**Figure 3:**
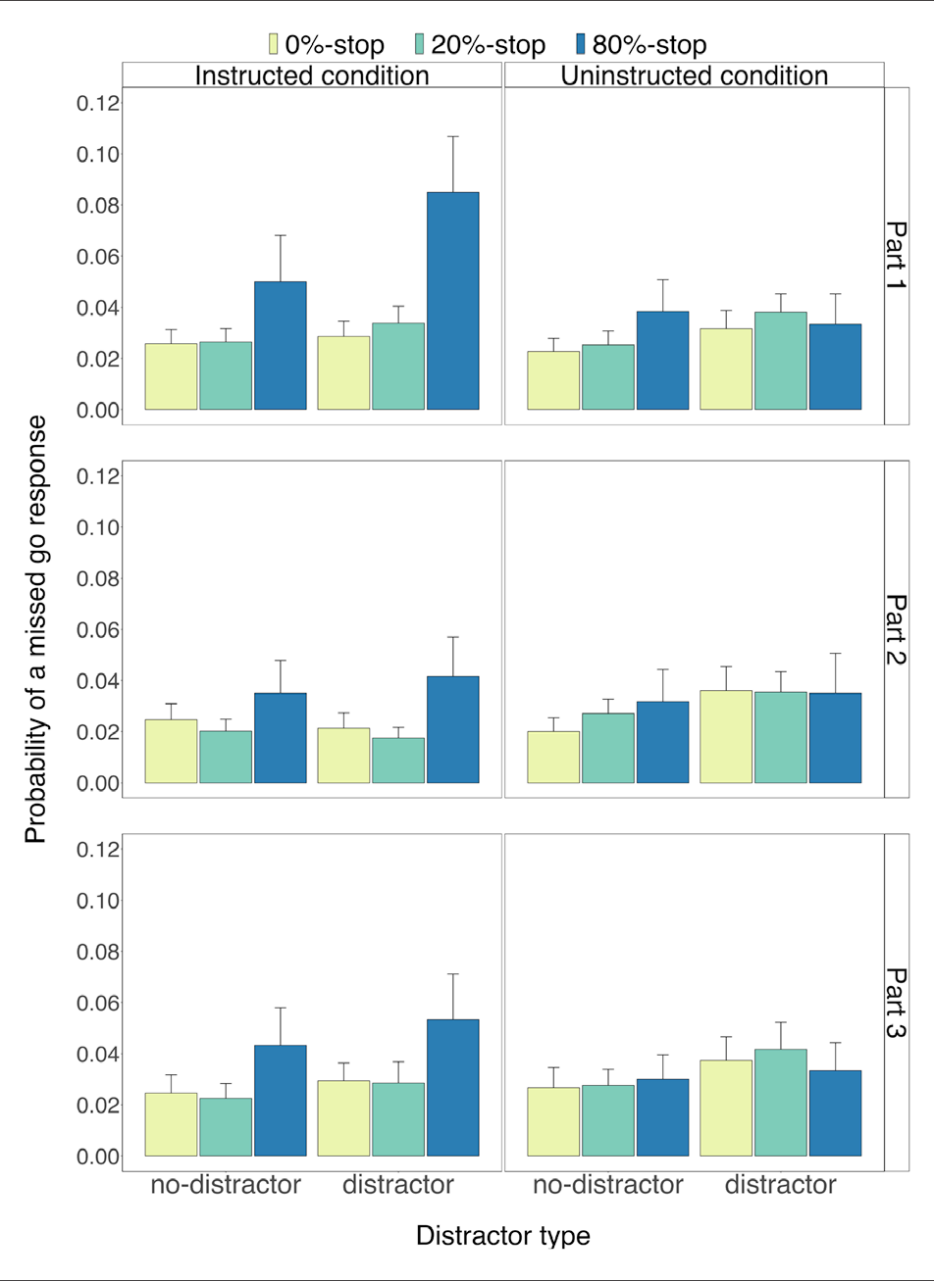
The probability of missed go responses for the instructed condition (left panels) and for the uninstructed condition (right panels) as a function of part (1–3), word type (0%-stop, 20%-stop or 80%-stop words) and distractor type (distractor, no-distractor). Error bars are 95% confidence intervals.

**Figure 4:**
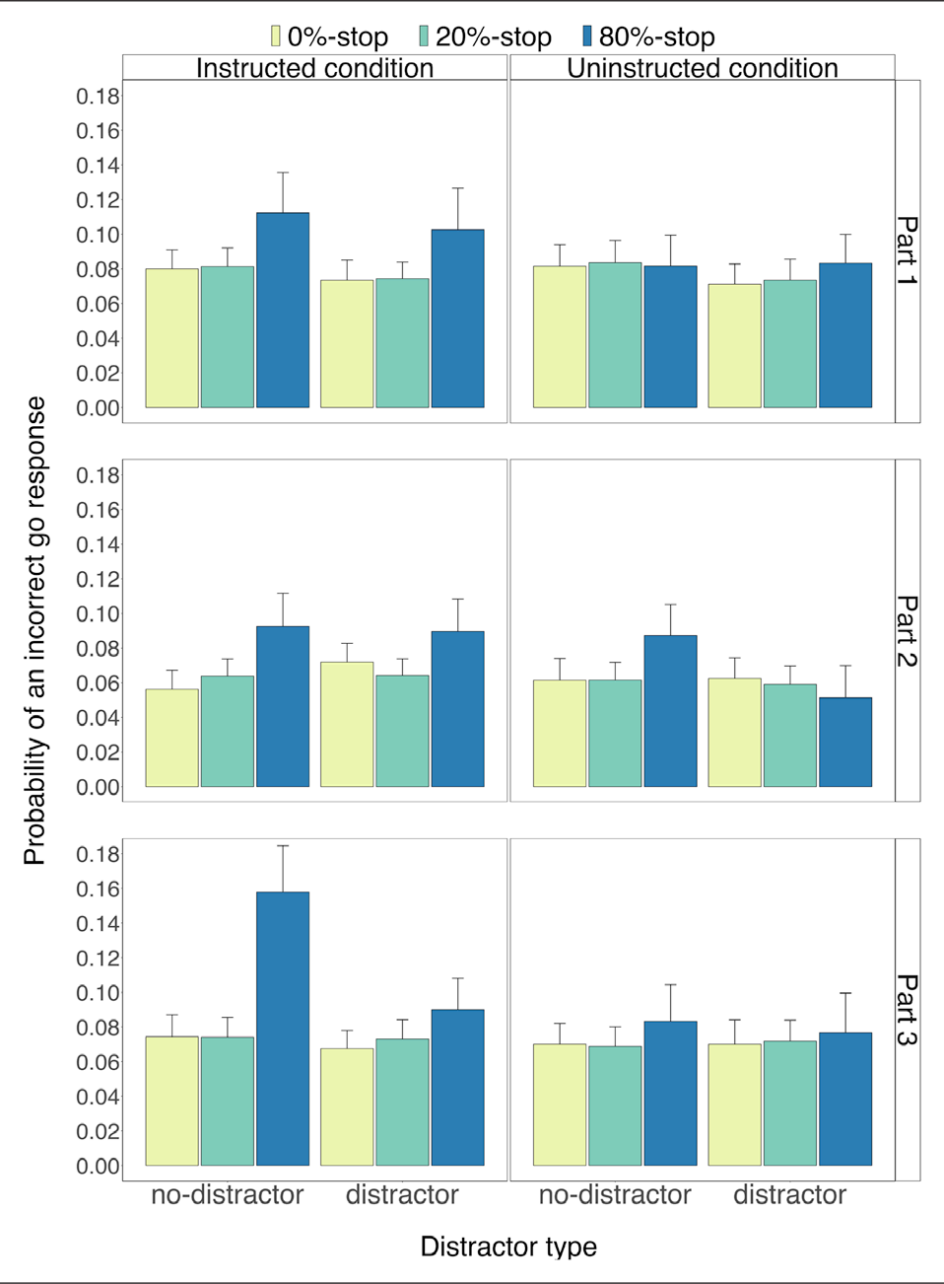
The probability of incorrect go responses for the instructed condition (left panels) and for the uninstructed condition (right panels) as a function of part (1–3), word type (0%-stop, 20%-stop or 80%-stop words) and distractor type (distractor, no-distractor). Error bars are 95% confidence intervals.

**Figure 5:**
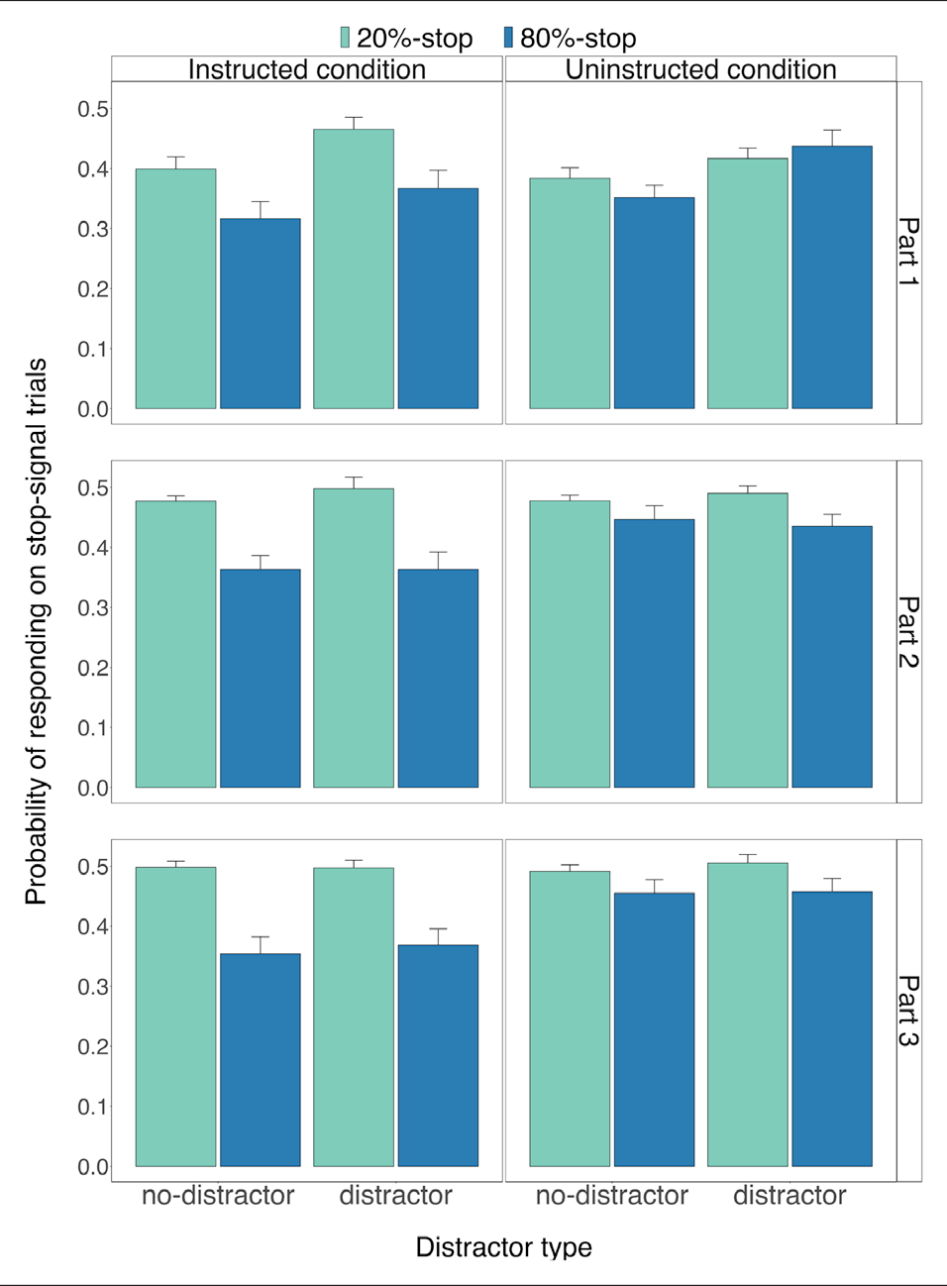
The probability of responding on stop-signal trials for the instructed condition (left panels) and for the uninstructed condition (right panels) as a function of part (1–3), word type (0%-stop, 20%-stop or 80%-stop words) and distractor type (distractor, no-distractor). Error bars are 95% confidence intervals.

Descriptive statistics appear in Figures 2, 3, 4, 5. Inferential statistics appear in Tables 1, 2, 3. Exploratory analyses of the eye-movement data of Experiment 1 and the behavioural analyses for each experiment separately are presented in the Supplementary Materials.

**Table 1:** Overview of the ANOVA conducted on the no-signal trial data. Condition is included as a between-subjects factor, all other factors are within-subjects. In the no-signal trial RT analysis, incorrect, and missed no-signal trials were removed.

	*Df1*	*Df2*	*Sum of squares effect*	*Sum of squares error*	*F*	*p*	*α_adj_*	*gen. η^2^*

**No-signal trials: RT**								
Condition	1	118	171051.50	94794587.10	0.21	0.645	0.017	0.002
Part	2	236	1783555.0	13010265.90	16.18	**<0.001**	0.004	0.016
Type	2	236	904922.00	966318.40	110.50	**<0.001**	0.004	0.008
Distract	1	118	1309571.0	896684.60	172.33	**<0.001**	0.003	0.011
Condition by part	2	236	64644.43	13010265.90	0.59	0.492	0.013	0.001
Condition by type	2	236	246795.90	966318.40	30.14	**<0.001**	0.004	0.002
Condition by distract	1	118	9326.65	896684.60	1.23	0.270	0.006	<0.001
Part by type	4	472	41534.56	1219546.70	4.02	0.011	0.005	<0.001
Part by distract	2	236	7617.38	664742.90	1.35	0.261	0.006	<0.001
Type by distract	2	236	3299.20	387996.50	1.00	0.349	0.007	<0.001
Condition by part by type	4	472	28190.83	1219546.70	2.73	0.052	0.005	<0.001
Condition by part by distract	2	236	812.94	664742.90	0.14	0.857	0.050	<0.001
Condition by type by distract	2	236	2398.44	387996.50	0.73	0.447	0.010	<0.001
Part by type by distract	4	472	7405.20	1006301.70	0.87	0.443	0.008	<0.001
Condition by part by type by distract	4	472	3603.93	1006301.70	0.42	0.705	0.025	<0.001
**No-signal trials: *p*(miss)**								
Condition	1	118	0.00	2.56	0.13	0.724	0.017	<0.001
Part	2	236	0.02	1.09	2.38	0.098	0.005	0.003
Type	2	236	0.10	0.66	18.03	**<0.001**	0.003	0.015
Distract	1	118	0.03	0.23	16.71	**<0.001**	0.004	0.005
Condition by part	2	236	0.02	1.09	2.02	0.138	0.006	0.003
Condition by type	2	236	0.07	0.66	11.92	**<0.001**	0.004	0.010
Condition by distract	1	118	0.00	0.23	0.03	0.869	0.050	<0.001
Part by type	4	472	0.02	1.07	1.89	0.146	0.006	0.003
Part by distract	2	236	0.00	0.28	1.22	0.296	0.008	<0.001
Type by distract	2	236	0.00	0.37	0.14	0.760	0.025	<0.001
Condition by part by type	4	472	0.01	1.07	0.83	0.453	0.010	0.001
Condition by part by distract	2	236	0.01	0.28	3.34	0.039	0.005	0.001
Condition by type by distract	2	236	0.02	0.37	6.54	0.007	0.004	0.003
Part by type by distract	4	472	0.00	0.55	0.59	0.585	0.013	<0.001
Condition by part by type by distract	4	472	0.01	0.55	1.37	0.254	0.007	0.001
**No-signal trials: *p*(error)**								
Condition	1	118	0.07	8.00	0.99	0.321	0.013	0.004
Part	2	236	0.10	1.13	9.95	**<0.001**	0.004	0.006
Type	2	236	0.23	1.38	19.83	**<0.001**	0.003	0.015
Distract	1	118	0.04	0.40	10.57	**0.001**	0.004	0.002
Condition by part	2	236	0.01	1.13	0.69	0.499	0.025	<0.001
Condition by type	2	236	0.10	1.38	8.39	**0.002**	0.004	0.007
Condition by distract	1	118	0.00	0.40	0.34	0.562	0.050	<0.001
Part by type	4	472	0.02	1.43	1.55	0.204	0.007	0.001
Part by distract	2	236	0.01	0.70	1.35	0.260	0.010	0.001
Type by distract	2	236	0.04	0.64	7.35	**0.003**	0.005	0.003
Condition by part by type	4	472	0.01	1.43	0.94	0.417	0.017	0.001
Condition by part by distract	2	236	0.04	0.70	6.33	**0.002**	0.005	0.003
Condition by type by distract	2	236	0.01	0.64	1.35	0.257	0.008	<0.001
Part by type by distract	4	472	0.04	1.24	3.56	0.019	0.006	0.002
Condition by part by type by distract	4	472	0.03	1.24	3.18	0.030	0.006	0.002

Note: distract = distractor type; type = word type. *α_ad_* = alpha-level following the sequential Bonferroni procedure to control for multiplicity. *p*s < *α_adj_* are highlighted in bold.

**Table 2:** No-signal trial RT Bayesian analysis. Bayes factors < 1 indicate that the removal of the factor or interaction had a deleterious effect on the model, whereas Bayes factors > 1 indicate that the factor or interaction could be removed without impairing the fit much.

Omitted factor(s)	Bayes Factor	Confidence interval

**Main analysis**		
Part	<0.00	±104.9%
Distract	<0.00	±66.33%
Type	<0.00	±89.32%
Condition by type	0.01	±98.86%
Condition	6.76	±65.95%
Condition by part	68.63	±103.7%
Condition by distract by part by type	268.60	±57.32%
Condition by distract by type	387.73	±91.84%
Condition by distract	702.78	±108.94%
Distract by part by type	796.09	±63.71%
Part by type	878.80	±88.37%
Distract by type	901.37	±88.94%
Condition by part by type	4387.12	±79.46%
Distract by part	4814.11	±97.42%
Condition by distract by part	88222.89	±108.29%

Note that ‘participant’ was included as a factor for all models, but this factor is not added to the model descriptions in the tables to reduce the amount of text. distract = distractor type; type = word type.

**Table 3:** Overview of the ANOVA conducted on the stop-signal data. Condition is included as a between-subjects factor, all other factors are within-subjects.

	*Df1*	*Df2*	*Sum of squares effect*	*Sum of squares error*	*F*	*p*	*α_adj_*	*gen. η^2^*

**Main analysis**								
Condition	1	118	0.36	5.35	7.94	0.006	0.006	0.019
Part	2	236	1.04	4.51	27.13	**<0.001**	0.004	0.053
Type	1	118	1.95	2.27	101.58	**<0.001**	0.003	0.095
Distract	1	118	0.21	0.99	24.50	**<0.001**	0.005	0.011
Condition by part	2	236	0.09	4.51	2.31	0.109	0.008	0.005
Condition by type	1	118	0.68	2.27	35.21	**<0.001**	0.004	0.035
Condition by distract	1	118	0.00	0.99	0.07	0.792	0.013	<0.001
Part by type	2	236	0.12	1.85	7.59	**0.001**	0.005	0.006
Part by distract	2	236	0.22	1.60	16.03	**<0.001**	0.004	0.012
Type by distract	1	118	0.00	0.73	0.00	0.947	0.050	<0.001
Condition by part by type	2	236	0.00	1.85	0.20	0.834	0.017	<0.001
Condition by part by distract	2	236	0.00	1.60	0.17	0.840	0.025	<0.001
Condition by type by distract	1	118	0.00	0.73	0.55	0.459	0.010	<0.001
Part by type by distract	2	236	0.03	1.36	2.26	0.106	0.007	0.001
Condition by part by type by distract	2	236	0.04	1.36	3.15	0.045	0.006	0.002

Note: distract = distractor type; type = word type. *α_ad_* = alpha-level following the sequential Bonferroni procedure to control for multiplicity. *p*s < *α_adj_* are highlighted in bold.

## Results

### No-signal analyses

***Reaction times on correct no-signal trials.*** Figure 2 shows correct RTs on no-signal trials for each condition, word type, distractor type, and part. Consistent with our predictions, we found a main effect of word type (*p* < 0.001; Table 1), and an interaction between condition and word type (*p* < 0.001; Table 1): the word-type effect was more pronounced in the instructed condition than in the uninstructed condition (Figure 2). Follow-up tests revealed that, as predicted, RTs on no-signal trials in the instructed condition were longer for the 80%-stop words (933 ms) than for the 0%-stop words (863 ms), *t*(59) = 10.17, *p* < 0.001, *g_av_* = 0.34, *BF_10_* = 519120863260 (one-tailed directional t-test: *p* < 0.001),^3^ and for the 20%-stop words (874 ms), *t*(59) = 9.34, *p* < 0.001, *g_av_* = 0.29, *BF_10_* = 25543245982 (one-tailed directional t-test: *p* < 0.001). The difference between the 0%-stop words and the 20%-stop words was also reliable, *t*(59) = –5.10, *p* < 0.001, *g_av_* = –0.05, *BF_10_* = 4510.62 (one-tailed directional t-test: *p* < 0.001). In the uninstructed condition, RTs on no-signal trials were longer for the 80%-stop words (921 ms) than for the 0%-stop words (900 ms), *t*(59) = 4.87, *p* < 0.001, *g_av_* = 0.10, *BF_10_* = 2095.25 (one-tailed directional t-test: *p* < 0.001), and for the 20%-stop words (902 ms), *t*(59) = 4.71, *p* < 0.001, *g_av_* = 0.09, *BF_10_* = 1197.50 (one-tailed directional t-test: *p* < 0.001). The difference between the 0%-stop words and the 20%-stop words was not reliable, *t*(59) = –1.14, *p* = 0.261, *g_av_* = –0.01 (one-tailed directional t-test: *p* = 0.130), *BF_10_* = 0.26.^4^ The correlations between the slowing for the 80%-stop words and the expectancy ratings in the instructed and uninstructed conditions will be discussed below (see ‘Expectancy analyses’).

Furthermore, as shown in Figure 2, the slowing for the 80%-stop words become numerically larger across task practice in both the instructed and uninstructed conditions indicating that learning took place in both conditions. Note, however, that the two-way interaction between word type and part and the three-way interaction between word type, part and condition did not survive correction for multiplicity.

Consistent with the earlier Verbruggen, Stevens et al. (2014) study, we found a main effect of distractor type (*p* < 0.001; Table 1). Importantly, however, there was no reliable difference in the magnitude of the distractor effect between conditions (*p* = 0.270; Table 1). Crucially, there was no reliable two-way interaction between word type and distractor type (*p* = 0.349; Table 1), nor a reliable three-way interaction between condition, word type, and distractor type (*p* = 0.447; Table 1). These conclusions were further supported by the Bayesian analyses. As can be seen in Table 2, the results of the Bayesian analyses are largely consistent with the ANOVAs reported in Table 1. The Bayesian analyses showed that dropping word type, distractor type, part, and the two-way interaction between condition and word type had a deleterious effect on the model fit. Importantly, the two-way interaction between word type and distractor type and the three-way interaction between condition, word type, and distractor type could be dropped. These analyses provide further support for the conclusion that the distractor effect was similar across the word types.

***Probability of a missed go response.*** Figure 3 shows the *p*(miss) data. We found a main effect of word type (*p* < 0.001; Table 1) and a reliable two-way interaction between condition and word type (*p* < 0.001; Table 1). Follow up tests revealed that there was a main effect of word type in the instructed condition (*p* < 0.001): *p*(miss) in the instructed condition was higher for the 80%-stop words (0.051) than for the 0%-stop words (0.026), *t*(59) = 4.43, *p* < 0.001, *g_av_* = 0.58, *BF_10_* = 493.45 (one-tailed directional t-test: *p* < 0.001), and for the 20%-stop words (0.025), *t*(59) = 4.43, *p* < 0.001, *g_av_* = 0.61, *BF_10_* = 495.29 (one-tailed directional t-test: *p* < 0.001). There was no reliable main effect of word type in the uninstructed condition (*p* = 0.224).

*P*(miss) was higher on distractor trials than on no-distractor trials (*p* < 0.001; Table 1). However, the two-way interaction between word type and distractor type was not reliable (*p* = 0.760; Table 1) and the three-way interaction between condition, word type, and distractor type did not survive after correction for multiplicity (*p* = 0.007; Table 1).

Combined, these results suggest that the instructed stimulus-stop associations increased outright stopping (or induced extreme slowing) following the presentation of 80%-stop words even when a go response was required. However, there was no evidence that the distractor effect varied across word types, thus replicating the results for RTs on no-signal trials.

***Probability of an incorrect go response.*** Figure 4 shows the *p*(error) data. Exploratory analyses of the *p*(error) data revealed a reliable main effect of word type (*p* < 0.001; Table 1) and a reliable two-way interaction between condition and word type (*p* < 0.001; Table 1). Follow-up tests revealed that the main effect of word type was reliable in the instructed condition (*p* < 0.001): error rates were higher for the 80%-stop words (0.11) than for the 0%-stop words (0.07), *t*(59) = 4.73, *p* < 0.001, *g_av_* = 0.53, *BF_10_* = 1289.83, and for the 20%-stop words (0.07), *t*(59) = 4.88, *p* < 0.001, *g_av_* = 0.51, *BF_10_* = 2113.55. There was no reliable difference between the 0%-stop words and the 20%-stop words, *t*(59) = –0.44, *p* = 0.661, *g_av_* = –0.03, *BF_10_* = 0.15. There was a similar numerical trend in the uninstructed condition (Figure 4, right panel), but there was no reliable main effect of word type (all *p* = 0.204) so pairwise comparisons between word types were not conducted.

There was a small but statistically significant difference between the no-distractor trials and the distractor trials (*p* = 0.001; Table 1). This effect did not interact with condition. The three-way interaction between word type, condition, and distractor was also not significant.

**Signal analyses.** Analyses of the probability of responding on stop-signal trials revealed, as predicted, a main effect of word type (20%-stop vs. 80%-stop words), *p* < 0.001 (Figure 5; Table 3). There was also a reliable two-way interaction between condition and word type (*p* < 0.001; Table 3) reflecting the larger difference between the 80%-stop words and the 20%-stop words in the instructed condition than in the uninstructed condition (Figure 5). Follow-up tests revealed that *p*(respond|signal) was lower for the 80%-stop words than for the 20%-stop words in the instructed condition (80%-stop words: 0.36, 20%-stop words: 0.47; *t*(59) = –9.86, *p* < 0.001, *g_av_* = –1.41, *BF_10_* = 170072648038) and in the uninstructed condition (80%-stop words: 0.43, 20%-stop words: 0.46; *t*(59) = –3.55, *p* = 0.001, *g_av_* = –0.57, *BF_10_* = 34.31). Thus, consistent with our hypothesis, stopping benefited from the stimulus-stop associations in the instructed and uninstructed conditions. Furthermore, the SSD values were similar for the 80%-stop and 20%-stop words (for descriptive statistics, see Supplementary Materials) due to the yoked tracking procedure (for details see *Procedure*). Thus, the difference in *p*(respond|signal) was not due to differences in the SSD.

Despite the separate tracking procedures, *p*(respond|signal) was lower on no-distractor trials than on distractor trials (*p* < 0.001; Table 3). This is consistent with our previous study which showed that distractors had a large effect on reactive inhibition (Verbruggen, Stevens, et al., 2014). Importantly, there was no reliable two-way interaction between word type and distractor type (interaction effect: *p* = 0.947, Table 3), and follow-up tests showed that the presence of distractors increased *p*(respond|signal) in the instructed condition (no-distractor: 0.40, distractor: 0.43; *t*(59) = 3.49, *p* = 0.001, *g_av_* = 0.31, *BF_10_* = 29.10) and in the uninstructed condition (no-distractor: 0.43, distractor: 0.46; *t*(59) = 3.52, *p* = 0.001, *g_av_* = 0.45, *BF_10_* = 31.45). The two-way interaction between part and distractor type was reliable (*p* < 0.001; Table 3); presumably, the separate tracking procedures were slowly ‘catching up’, reducing the distractor difference over blocks.

**Figure 6:**
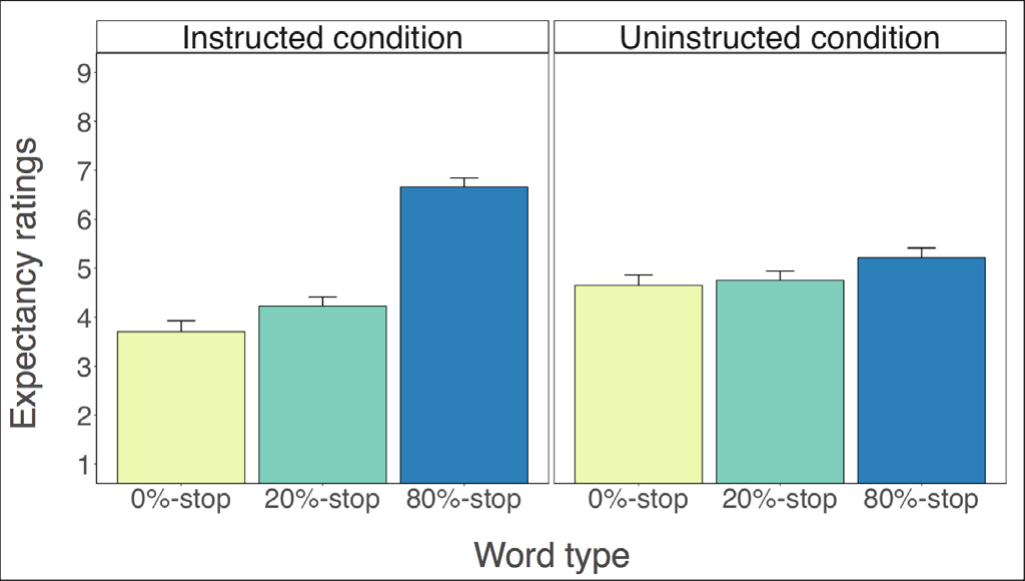
Expectancy ratings for the instructed condition (left panels) and for the uninstructed condition (right panels) as a function of word type (0%-stop, 20%-stop or 80%-stop words). Note: 1 = ‘*I definitely do not think this word indicates that I have to withhold my response*’; 9 = ‘*I definitely think this word indicates that I have to withhold my response*’. Error bars are 95% confidence intervals.

**Expectancy analyses.** Analyses of the expectancy data revealed a main effect of word type, *F*(2, 236) = 142.24, *p* < 0.001, *gen. η^2^* = 0.329 (Figure 6). There was also a reliable interaction between word type and condition, *F*(2, 236) = 65.15, *p* < 0.001, *gen. η^2^* = 0.183, indicating that the differences between the word types were larger in the instructed condition than in the uninstructed condition.

Consistent with the stimulus-stop contingencies, planned comparisons revealed that participants in the instructed condition expected to stop more for the 80%-stop words than for the 0%-stop words, *t*(59) = –12.98, *p* < 0.001, *g_av_* = 2.62, *BF_10_* = 8.67e+15, and for the 20%-stop words, *t*(59) = –11.27, *p* < 0.001, *g_av_* = 2.35, *BF_10_* = 2.56e + 13 (Figure 6).^5^ This is consistent with our hypothesis. The difference between the 0%-stop words and the 20%-stop words in the instructed condition was also reliable, *t*(59) = –5.47, *p* < 0.001, *g_av_* = –0.46, *BF_10_* = 16531.78, mirroring a similar difference in the task performance data for RTs and errors on no-signal trials. In other words, extra learning occurred in the instructed condition. It is possible that informing participants in the instructed condition about the 80%-stop words influenced both the strength and/or quality of their knowledge representations regarding the existence of stimulus-stop contingencies in the task. Participants in the uninstructed condition also expected to stop more for the 80%-stop words than for the 0%-stop words, *t*(59) = –4.26, *p* < 0.001, *g_av_* = 0.50, *BF_10_* = 287.76, and for the 20%-stop words, *t*(59) = –4.01, *p* < 0.001, *g_av_* = 0.44, *BF_10_* = 131.54.^6^ The difference between the 0%-stop words and the 20%-stop words in the uninstructed condition was not reliable, *t*(59) = –1.03, *p* = 0.307, *g_av_* = –0.09, *BF_10_* = 0.23. Thus, participants in both conditions could distinguish between the 80%-stop words and the other word types on the basis of their association with stopping.

**Figure 7:**
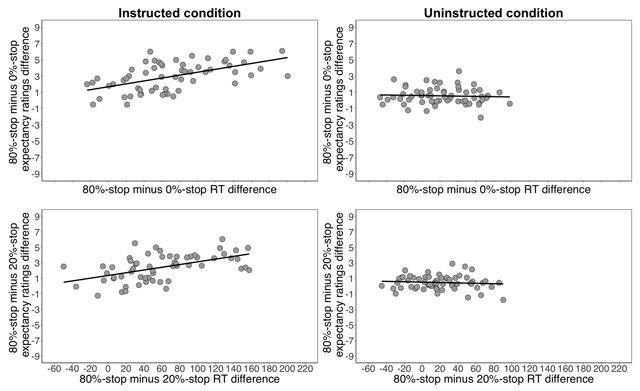
Expectancy/RT correlations for the 80%-stop and 0%-stop words (upper panels) and the 80%-stop and 20%-stop words (lower panels) for the instructed condition (left panels) and for the uninstructed condition (right panels). The correlations in the instructed condition were reliable (*p*s ≤ 0.001) but the correlations in the uninstructed condition were not reliable (*p*s ≥ 0.501).

Next we investigated the relationship between the expectancy ratings and task performance (Figure 7). In the instructed condition, as predicted, the 80%-stop vs. 0%-stop expectancy difference reliably correlated with the corresponding RT difference, *r*(58) = 0.548, *p* < 0.001; similarly, the 80%-stop vs. 20%-stop expectancy difference reliably correlated with the corresponding RT difference, *r*(58) = 0.524, *p* < 0.001. In the uninstructed condition, again as predicted, both correlations were not significant (*r*s ≤ –0.09, *p*s ≥ 0.501; note that uncorrected *p*s are reported). To investigate whether the correlations in the instructed and uninstructed conditions were statistically different, we compared the correlation coefficients in the instructed and uninstructed conditions using the Fisher’s r-to-z transformation. This confirmed that the difference between the instructed and uninstructed conditions was reliable for the 80% vs. 0%-stop correlations (*Z* = 3.59, *p* < 0.001) and for the 80% vs. 20%-stop correlations (Z = 3.58, *p* < 0.001). To quantify the evidence in favour of the null hypothesis (of no relationship) and of the alternative hypothesis (of a relationship), we also conducted Bayesian regression analyses. These analyses supported the null hypothesis that the expectancy ratings did not correlate with the RT slowing in the uninstructed condition (*BF* 80%-stop vs. 0%-stop words: 0.28; *BF* 80%-stop vs. 20%-stop words: 0.32), whereas the same analyses supported the alternative hypothesis for the instructed analysis (*BF* 80%-stop vs. 0%-stop words: 2701.52; *BF* 80%-stop vs. 20%-stop words: 1023.85).

Inspection of Figure 7 shows that the difference between these correlations is unlikely to be entirely due to differences in the range of the RT and/or expectancy values between the instructed and uninstructed conditions. However, to test this possibility further, we excluded RT-difference scores (and corresponding expectancy-difference scores) in the instructed condition that were less than or greater than the lowest and highest RT difference scores, respectively, in the uninstructed condition. There were 44 participants remaining for the 80%-stop vs. 0%-stop correlation and 46 participants for the 80%-stop vs. 20%-stop correlation. Nevertheless, the observed correlations in the instructed condition remained reliable and the Bayes Factors showed substantial support for the alternative hypothesis (*r*s ≥ 0.385, *p*s ≤ 0.010, BFs ≥ 4.99). Furthermore, this difference cannot be easily explained by the differences in the acquisition rates of the stimulus-stop contingencies between the instructed and uninstructed conditions; RT/expectancy correlations performed on only part 3 (i.e. the final part of training) showed a similar pattern as the aforementioned correlations with all parts of training included. In the instructed condition, we found that the 80%-stop vs. 0%-stop expectancy difference reliably correlated with the corresponding RT difference, *r*(58) = 0.419, *p* < 0.001; similarly, the 80%-stop vs. 20%-stop expectancy difference reliably correlated with the corresponding RT difference, *r*(58) = 0.341, *p* = 0.008. In the uninstructed condition, all correlations were not significant (*p*s ≥ 0.223). Thus, it seems that although the behavioural effects are the same (i.e. response slowing for 80%-stop words), there is good evidence that the underlying mechanisms are somewhat different for the instructed and uninstructed conditions.

## General Discussion

Responding is typically slowed for items that were previously associated with stopping. This slowing has been attributed to the retrieval of stimulus-stop associations which ‘directly’ activate the stop goal/response and slow responding on no-signal trials. Thus, learning could reduce the need for reactive and instruction-based control in response-inhibition tasks as the stop process could be activated before an extra signal is presented (Verbruggen, Best, et al., 2014; Verbruggen & Logan, 2008b). However, in some recent experiments we found that slowing for old stop words was mediated by expectancies of the stimulus-stop contingencies acquired during training (Best et al., 2016). Previous work has demonstrated that when an instructional cue [e.g. ‘*p*(stop-signal) = .75’] indicates that a stop signal is likely to occur on the following trial(s), participants slow down by proactively adjusting their processing strategies (for reviews, see e.g. Aron, 2011; Verbruggen & Logan, 2017). Therefore, if participants use stop-associated items as similar cues, slowing for old stop-associated words could reflect proactive (anticipatory) strategy adjustments driven by expectancies of the stimulus-stop contingencies in play rather than some more direct activation of the stop process. In the present study we aimed to test this hypothesis using a combination of an instruction and distractor manipulation. We also measured expectancy ratings following task completion (as in our previous study; Best et al., 2016).

### Similarities between the instructed and uninstructed conditions

Consistent with our predictions, our results show that the stimulus-stop contingencies influenced task performance in both the instructed and the uninstructed conditions: RTs on no-signal trials were higher and the *p*(respond|signal) was lower for the stop-associated words (80%-stop) than for the go-associated (0%-stop) words and for the control (20%-stop) words. Consistent with our instruction manipulation and predictions, the effects of word type on performance were greater in the instructed condition (where participants were told beforehand which words would likely be associated with a stop signal) than in the uninstructed condition (where participants received no such information). This difference remained relatively stable over time, as indicated by the absence of a three-way interaction between part, word type, and condition.

Participants in both conditions also generated expectancies that were consistent with the stimulus-stop contingencies in play: they expected to stop their responses more when stop-associated words were presented than when go-associated or control words were presented.

Combined, these results suggest that stimulus-stop associations acquired primarily through explicit instructions and uninstructed stimulus-stop associations acquired primarily through task practice have similar effects on task performance (i.e. both induce slowing on no-signal trials and reduce the probability of responding on stop-signal trials). However, the effects are much stronger when the stop contingencies are instructed.

The overlap between the instructed and uninstructed conditions is consistent with previous work that has demonstrated that key areas of the instruction-based inhibitory control network are activated following the presentation of old stop-associated items (e.g. Lenartowicz et al., 2011). Furthermore, it has been argued that despite the differences in learning speed, similar learning mechanisms may underlie instruction- or rule-based behaviour and stimulus-response link based behaviour (e.g. Pfeuffer, Hosp, Kimmig, Moutsopoulou, Waszak, & Kiesel, 2017; Pfeuffer, Moutsopoulou, Pfister, Waszak, & Kiesel, 2017; Pfeuffer, Moutsopoulou, Waszak, & Kiesel, 2018; Ramamoorthy & Verguts, 2012; Verbruggen, McLaren, et al., 2014). Given this overlap between instruction- and practice-based inhibition, it is perhaps unsurprising that we find similarities between the two instruction conditions in the present study.

### Differences between the instructed and uninstructed conditions

Despite the overlap, it is noteworthy that extra practice did not reduce the difference between the two instruction conditions. Thus, the difference between the instructed and uninstructed condition may not be (only) due to slower learning rates in the latter. Indeed, we also found some other differences between the instructed and uninstructed conditions. In the instructed condition, participants were slower to respond to the 20%-stop words during task performance and expected to stop more to the 20%-stop words compared with the 0%-stop words. We found no reliable differences between the 20%-stop and 0%-stop words in the uninstructed condition. This suggests that instructing the participants about the 80%-stop words influenced their meta-knowledge about the existence of stimulus-stop contingencies in the task and encouraged them to look for others (“I know there are words often paired with the stop-signal – it might be good to search for other structures in the task material as well”, cf. Gaschler et al., 2014), alongside influencing their knowledge about specific 80%-stop words (“I know that this word is often paired with stop”).

Furthermore, in the instructed condition, we found that the expectancy ratings correlated with task performance: participants who expected to withhold their response more for the 80%-stop words responded more slowly to these words than to the 0%-stop words and to the 20%-stop words. In contrast, the expectancy ratings in the uninstructed condition did not reliably correlate with task performance. This conclusion was further supported by Bayesian regression analyses. In other words, participants could generate expectancies (at least at the end of the experiment), but these expectancies did not seem to influence task performance.

In Best et al. (2016), we also found a correlation between slowing and expectancy ratings, even though all stimulus-stop mappings were uninstructed. As noted in the Introduction, correlations between expectancy ratings and performance are consistent with the idea that slowing reflects strategic proactive control adjustments, whereas the absence of a correlation is more consistent with the idea that the retrieval of stop associations directly interferes with responding. Differences in task characteristics (e.g. in Best et al., we used a hybrid go/no-go task whereas we used a stop-signal task in the present study) could have influenced what was learned and how the acquired associations influenced subsequent performance in our two studies. It is also possible, however, that expectancies simply constitute a measure of the strength of stimulus-stop learning on performance, in which case the difference between studies is quantitive (i.e. the amount of learning) rather than qualitative (strategy adjustments vs. direct activation).

It is also important to note that the association between expectancies and task performance was correlational and not causal. To establish a causal role of explicit expectancies and to further disentangle contributions of expectancies and learning in response-inhibition tasks, future research could, for example, instruct expectations that do not fully match with the contingencies in the task material (Biele, Rieskamp, Gonzalez, 2009; Doll, Jakobs, Sanfey, Frank, 2009; Umbach, Schwager, Frensch, & Gaschler, 2012).

### No interactions with the distractor effect

In Verbruggen, Stevens, et al. (2014), expectancy was manipulated in a block-based fashion (i.e. participants were informed at the beginning of a block if signals could occur in the periphery or in the centre of the screen), and strategic proactive control adjustments increased the distractor effect in RTs on no-signal trials. We used the same distractor manipulation in the present study to distinguish the roles of strategic proactive control and direct retrieval (of the stimulus-stop associations) in a stop-learning task. Consistent with Verbruggen, Stevens, et al. (2014), we predicted that participants in instructed condition would proactively adjust their attention when presented with 80%-stop words; consequently, the distractor effect would be larger for the 80%-stop words compared with the other word types. Furthermore, if participants in the uninstructed condition similarly used the 80%-stop words as cues to elicit proactive attentional control, we would see a larger distractor effect for these words emerge over task practice in this condition as well. In contrast, the ‘direct retrieval’ account predicted no change in the distractor effect across word types (if anything, the effect could become smaller because there is less need to monitor for stop signals if the word is highly predictive of a signal). Thus, we anticipated that the inclusion of the distractor manipulation would allow us to determine the involvement of strategic proactive control adjustments during stop learning.

Contrary to our predictions, we found that word-specific stop learning did not interact with the distractor effect on no-signal RTs. Importantly, this was the case in both the instructed and uninstructed conditions (and in the uninstructed condition of an earlier pilot experiment; see Supplementary Materials). Here, we suggest three of the most plausible alternative explanations for the absence of an interaction between expectancy and the distractor manipulation in no-signal RTs.

First, it is possible that participants simply cannot adjust their attentional settings on a trial-by-trial basis (see Strayer & Kramer, 1994, for a similar idea). However, evidence in the task-switching literature has demonstrated that participants can reconfigure their attentional settings when a cue indicates that a task switch is required (e.g. Longman, Lavric, & Monsell, 2013; Longman, Lavric, Munteanu, & Monsell, 2014; Rushworth, Passingham, & Nobre, 2005). Furthermore, trial-by-trial changes in attentional settings were also observed in the proactive inhibitory control study of Elchlepp et al. (2016). Combined, these findings indicate that trial-by-trial adjustments in attentional settings can indeed be made (see also Verbruggen & Logan, 2009a).

Second, it is possible that in the previous study by Verbruggen, Stevens, et al. (2014), the presence of distractors primarily interfered with the detection or analysis of the go words rather than response selection. In the original experiment, two words were presented and participants had to respond to the location of the natural item, whereas only one word was presented in the present study. Thus, the attentional demands were higher in Verbruggen, Stevens, et al. (2014).

Third, we should also consider the possibility that the increased distractor effect in non-central signal blocks reported by Verbruggen, Stevens, et al. (2014) reflects a ‘false positive’. After we failed to observe an increased distractor effect in the instructed condition of the present study, we ran a further (unpublished) experiment with two words per trial. There were ‘pure’ blocks in which the location of the signal stayed the same (non-central signal blocks, central signal blocks, and no-signal blocks as in Verbruggen, Stevens, et al. 2014) and ‘mixed’ blocks in which the location of the signal changed on a trial-by-trial basis. In both block types, the location of the upcoming signal was instructed by way of a cue presented at the beginning of each trial. We did not find any differences in the size of the distractor effect across the signal locations in the pure blocks or in the mixed blocks. One possibility is that the presentation of the cue on each trial encouraged participants to attend to the centre of the screen in that experiment. Alternatively, it could be that attentional adjustments, if made, do not reliably increase the distractor effect in this stop-signal task after all.

We cannot currently distinguish between the different explanations, so future research is required here. Note that the present study found effects of distractors on reactive inhibition (as indexed by *p*(respond|signal)). We also replicated the distractor effect on reactive stopping in another recent study (Dodds et al., In Preparation). Thus, we consistently replicated the reactive inhibition part of the Verbruggen, Stevens, et al. (2014) findings. This difference between reactive and proactive inhibition is not surprising, as distractors had a much larger effect on reactive control than proactive control in the original study (Verbruggen, Stevens, et al., 2014). To detect attentional strategy adjustments, more sensitive measures might be needed (such as EEG; see Elchlepp et al., 2016; Schevernels et al., 2015).

## Conclusion

In conclusion, these results suggest that slowing for stimuli that are consistently associated with stopping can arise from both instructed and acquired mappings. Given that we found effects of the stimulus-stop contingencies on expectancies in both the instructed and uninstructed conditions, the most parsimonious explanation would be to argue that explicit knowledge is required to observe response slowing for old stop items. However, Bayesian analyses showed that expectancies correlated with response slowing on no-signal trials in the instructed condition but not in the uninstructed condition. Thus, the present study provides some evidence that stimulus-stop learning may well be mediated via both expectancies and information acquired via experience.

## Additional File

The additional file for this article can be found as follows:

10.5334/joc.53.s1 Supplementary Material. Descriptive and inferential statistics for Experiment 1 and 2.

## Data Availability

All data files and analysis scripts are deposited in the Open Science Framework (https://osf.io/kydw6/?view_only=131cbcc73fce4abfbfc34375819ecaad).

## Footnotes

‘Automaticity’ in this account is defined on the basis of Instance Theory (Logan, 1988). In this theory, performance was considered to be ‘automatic’ when it was based on the retrieval of previous instances or episodes from memory.

Performance on distractor trials minus performance on no-distractor trials.

Based on previous evidence of stimulus-stop learning, we made strong predictions about the direction of this effect. No differences between word types or differences in the opposite direction (i.e. shorter RTs or lower *p*(miss) for 80% words than for 0%-stop and 20%-stop words) would argue against stimulus-stop associations. Therefore, we report both the two-tailed *p*-values and the *p*-values of one-directional t-tests.

To explore the difference between the 0%-stop words and the 20%-stop words we repeated the main analysis excluding the 80%-stop words. This showed that the two-way interaction between condition and word type was reliable, *F*(1, 118) = 7.12, *p* = 0.009, *gen. η^2^* < 0.001, reflecting the larger RT differences between the 0%-stop words and the 20%-stop words in the instructed condition than in the uninstructed condition. Furthermore, the three way interaction between condition, word type and part was also reliable, *F*(2, 236) = 3.07, *p* = 0.047, *gen. η^2^* < 0.001: the numerical difference between the 0%-stop words and the 20%-stop words became larger as a function of part in the instructed condition but became smaller as a function of part in the uninstructed condition.

In the Instructed condition, 97% of participants expected to stop more for the 80%-stop words than for the 0%-stop words and 90% of participants expected to stop more for the 80%-stop words than for the 20%-stop words (90% expected to stop more for the 80%-stop items than the 0%-stop *and* 20%-stop words).

In the uninstructed condition, 67% of participants expected to stop more for the 80%-stop words than for the 0%-stop words and 70% of participants expected to stop more for the 80%-stop words than for the 20%-stop words (48% expected to stop more for the 80%-stop items than the 0%-stop *and* 20%-stop words).

## Author Information

Correspondence concerning this article should be addressed to Maisy Best or Frederick Verbruggen. MB: Institute of Neuroscience and Psychology, University of Glasgow, Glasgow, United Kingdom; FV: Experimental Psychology, Ghent University, Ghent, Belgium. E-mail: maisy.jane.best@gmail.com or frederick.verbruggen@ugent.be. We thank Tobias Stevens for help with a pilot study. This work was supported by an Economic and Social Research Council Grant (ES/J00815X/1) to FV and IPLM, a studentship from the Economic and Social Research Council to MB (ES/J50015X/1), and a starting grant to FV from the European Research Council (ERC) under the European Union’s Seventh Framework Programme (FP7/2007-2013)/ERC Grant Agreement No. 312445. FV is currently supported by an ERC Consolidator grant (European Union’s Horizon 2020 Research and Innovation Programme, Grant Agreement No. 769595).

## References

[1] Adams, R. C., Lawrence, N. S., Verbruggen, F., & Chambers, C. D. (2017). Training response inhibition to reduce food consumption: Mechanisms, stimulus specificity and appropriate training protocols. Appetite, 109, 11–23. DOI: 10.1016/j.appet.2016.11.014 27838443 PMC5240656

[2] Allom, V., Mullan, B., & Hagger, M. (2015). Does inhibitory control training improve health behaviour? A meta-analysis. Health Psychology Review. DOI: 10.1080/17437199.2015.1051078 26058688

[3] Aron, A. R. (2011). From reactive to proactive and selective control: Developing a richer model for stopping inappropriate responses. Biological Psychiatry, 69, e55–68. DOI: 10.1016/j.biopsych.2010.07.024 20932513 PMC3039712

[4] Best, M., Lawrence, N. S., Logan, G. D., McLaren, I. P. L., & Verbruggen, F. (2016). Should we stop or should we go? The role of associations and expectancies. Journal of Experimental Psychology: Human Perception and Performance, 42, 115–137. DOI: 10.1037/xhp0000116 26322688 PMC4685931

[5] Best, M., & Verbruggen, F. (under review). Does learning influence the detection of signals in a response-inhibition task? Journal of Cognition. 10.5334/joc.73 PMC6676923 31517237

[6] Bestmann, S. (2012). Functional modulation of primary motor cortex during action selection In: Chen, R., & Rothwell, J. C. (Eds.), Cortical connectivity, 183–205. Springer DOI: 10.1007/978-3-662-45797-9_10

[7] Biele, G., Rieskamp, J., & Gonzalez, R. (2009). Computational models for the combination of advice and individual learning. Cognitive Science, 33(2), 206–242. DOI: 10.1111/j.1551-6709.2009.01010.x 21585468

[8] Bowditch, W. A., Verbruggen, F., & McLaren, I. P. (2016). Associatively mediated stopping: Training stimulus-specific inhibitory control. Learning & Behavior, 44(2), 162–174. DOI: 10.3758/s13420-015-0196-8 26400499

[9] Brainard, D. H. (1997). The psychophysics toolbox. Spatial Vision, 10, 433–6. DOI: 10.1163/156856897X00357 9176952

[10] Chambers, C. D., Garavan, H., & Bellgrove, M. A. (2009). Insights into the neural basis of response inhibition from cognitive and clinical neuroscience. Neuroscience and Biobehavioral Reviews, 33, 631–646. DOI: 10.1016/j.neubiorev.2008.08.016 18835296

[11] Chein, J. M., & Schneider, W. (2012). The Brain’s Learning and Control Architecture. Current Directions in Psychological Science, 21, 78–84. DOI: 10.1177/0963721411434977

[12] Chiu, Y. C., Aron, A. R., & Verbruggen, F. (2012). Response suppression by automatic retrieval of stimulus-stop association: Evidence from transcranial magnetic stimulation. Journal of Cognitive Neuroscience, 24, 1908–1918. DOI: 10.1162/jocn_a_00247 22624606 PMC4420637

[13] Cramer, A. O. J., van Ravenzwaaij, D., Matzke, D., Steingroever, H., Wetzels, R., Grasman, R. P. P. P., Waldorp, L. J., & Wagenmakers, E.-J. (2016). Hidden multiplicity in exploratory multiway ANOVA: Prevalence and remedies. Psychonomic Bulletin & Review, 23(2), 640–647. DOI: 10.3758/s13423-015-0913-5 26374437 PMC4828473

[14] Dodds, C. M., Howson, S., Stevens, T., Heinzel, C., Hobbs, M., Muller, U., Verbruggen, F., Morgan, C. J. A., & Zeman, A. (In Preparation). Effects of Methylphenidate on Stopping While Distracted.

[15] Doll, B. B., Jacobs, W. J., Sanfey, A. G., & Frank, M. J. (2009). Instructional control of reinforcement learning: A behavioral and neurocomputational investigation. Brain Research, 1299, 74–94. DOI: 10.1016/j.brainres.2009.07.007 19595993 PMC3050481

[16] Elchlepp, H., Lavric, A., Chambers, C. D., & Verbruggen, F. (2016). Proactive inhibitory control: A general biasing account. Cognitive Psychology, 86, 27–61. DOI: 10.1016/j.cogpsych.2016.01.004 26859519 PMC4825542

[17] Faul, F., Erdfelder, E., Buchner, A., & Lang, A. G. (2009). Statistical power analyses using G* Power 3.1: Tests for correlation and regression analyses. Behavior Research Methods, 41(4), 1149–1160. DOI: 10.3758/BRM.41.4.1149 19897823

[18] Gaschler, R., Marewski, J. N., Wenke, D., & Frensch, P. A. (2014). Transferring control demands across incidental learning tasks–stronger sequence usage in serial reaction task after shortcut option in letter string checking. Frontiers in Psychology, 5, 1388 DOI: 10.3389/fpsyg.2014.01388 25506336 PMC4246662

[19] Houben, K. (2011). Overcoming the urge to splurge: Influencing eating behavior by manipulating inhibitory control. Journal of Behavior Therapy and Experimental Psychiatry, 42, 384–388. DOI: 10.1016/j.jbtep.2011.02.008 21450264

[20] Houben, K., & Jansen, A. (2011). Training inhibitory control. A recipe for resisting sweet temptations. Appetite, 56, 345–349. DOI: 10.1016/j.appet.2010.12.017 21185896

[21] Houben, K., Nederkoorn, C., Wiers, R. W., & Jansen, A. (2011). Resisting temptation: Decreasing alcohol-related affect and drinking behavior by training response inhibition. Drug and Alcohol Dependence, 116 (1–3), 132–136. DOI: 10.1016/j.drugalcdep.2010.12.011 21288663

[22] Jahfari, S., Stinear, C. M., Claffey, M., Verbruggen, F., & Aron, A. R. (2010). Responding with restraint: What are the neurocognitive mechanisms? Journal of Cognitive Neuroscience, 22, 1479–92. DOI: 10.1162/jocn.2009.21307 19583473 PMC2952035

[23] Jones, A., Di Lemma, L. C. G., Robinson, E., Christiansen, P., Nolan, S., Tudur-Smith, C., & Field, M. (2016). Inhibitory control training for appetitive behaviour change: A meta-analytic investigation of mechanisms of action and moderators of effectiveness. Appetite, 97, 16–28. DOI: 10.1016/j.appet.2015.11.013 26592707

[24] Jones, A., & Field, M. (2013). The effects of cue-specific inhibition training on alcohol consumption in heavy social drinkers. Experimental and Clinical Psychopharmacology, 21, 8–16. DOI: 10.1037/a0030683 23181512

[25] Lakens, D. (2013). Calculating and reporting effect sizes to facilitate cumulative science: A practical primer for t-tests and ANOVAs. Frontiers in Psychology, 4, 863 DOI: 10.3389/fpsyg.2013.00863 24324449 PMC3840331

[26] Lawrence, N. S., O’Sullivan, J., Parslow, D., Javaid, M., Adams, R. C., Chambers, C. D., Kos, K., & Verbruggen, F. (2015a). Training response inhibition to food is associated with weight loss and reduced energy intake. Appetite, 95, 17–28. DOI: 10.1016/j.appet.2015.06.009 26122756 PMC4596151

[27] Lawrence, N. S., Verbruggen, F., Morrison, S., Adams, R. C., & Chambers, C. D. (2015b). Stopping to food can reduce intake. Effects of stimulus- specificity and individual differences in dietary restraint. Appetite, 85, 91–103. DOI: 10.1016/j.appet.2014.11.006 25447023 PMC4286116

[28] Lenartowicz, A., Verbruggen, F., Logan, G. D., & Poldrack, R. A. (2011). Inhibition-related activation in the right inferior frontal gyrus in the absence of inhibitory cues. Journal of Cognitive Neuroscience, 23, 3388–3399. DOI: 10.1162/jocn_a_00031 21452946

[29] Liefooghe, B., Degryse, J., & Theeuwes, M. (2016). Automatic effects of no-go instructions. Canadian Journal of Experimental Psychology, 70(3), 232–241. DOI: 10.1037/cep0000080 27077956

[30] Logan, G. D. (1988). Toward an instance theory of automatization. Psychological Review, 95, 492–527. DOI: 10.1037/0033-295X.95.4.492

[31] Longman, C. S., Lavric, A., & Monsell, S. (2013). More attention to attention? An eye-tracking investigation of selection of perceptual attributes during a task switch. Journal of Experimental Psychology: Learning, Memory, and Cognition, 39(4), 1142–1151. DOI: 10.1037/a0030409 23088543

[32] Longman, C. S., Lavric, A., Munteanu, C., & Monsell, S. (2014). Attentional inertia and delayed orienting of spatial attention in task-switching. Journal of Experimental Psychology: Human Perception and Performance, 40, 1580–1602. DOI: 10.1037/a0036552 24842065

[33] Meyer, D. E., & Kieras, D. E. (1997). A computational theory of executive cognitive processes and multiple-task performance: Part 1. Basic mechanisms. Psychological Review, 104, 3–65. DOI: 10.1037/0033-295X.104.1.3 9009880

[34] Miyake, A., Friedman, N. P., Emerson, M. J., Witzki, A. H., Howerter, A., & Wager, T. D. (2000). The unity and diversity of executive functions and their contributions to complex “frontal lobe” tasks: A latent variable analysis. Cognitive Psychology, 41, 49–100. DOI: 10.1006/cogp.1999.0734 10945922

[35] Morey, R. D., Rouder, J. N., & Jamil, T. (2015). BayesFactor: Computation of Bayes factors for common designs (Version 0.9.11-1). Retrieved from: https://cran.r-project.org/web/packages/BayesFactor/.

[36] Newell, B. R., & Shanks, D. R. (2014). Unconscious influences on decision making: A critical review. The Behavioral and Brain Sciences, 1–19. DOI: 10.1017/S0140525X12003214 24461214

[37] Noël, X., Brevers, D., Hanak, C., Kornreich, C., Verbanck, P., & Verbruggen, F. (2016). On the automaticity of response inhibition in individuals with alcoholism. Journal of Behavior Therapy and Experimental Psychiatry, 51, 84–91. DOI: 10.1016/j.jbtep.2016.01.003 26800080

[38] Pfeuffer, C. U., Hosp, T., Kimmig, E., Moutsopoulou, K., Waszak, F., & Kiesel, A. (2017). Defining stimulus representation in stimulus–response associations formed on the basis of task execution and verbal codes. Psychological Research, 1–15. DOI: 10.1007/s00426-017-0861-y 28391366

[39] Pfeuffer, C. U., Moutsopoulou, K., Pfister, R., Waszak, F., & Kiesel, A. (2017). The power of words: On item-specific stimulus–response associations formed in the absence of action. Journal of Experimental Psychology: Human Perception and Performance, 43(2), 328–347. DOI: 10.1037/xhp0000317 27831720

[40] Pfeuffer, C. U., Moutsopoulou, K., Waszak, F., & Kiesel, A. (2018). Multiple priming instances increase the impact of practice-based but not verbal code-based stimulus-response associations. Acta psychologica, 184, 100–109. DOI: 10.1016/j.actpsy.2017.05.001 28511771

[41] Ramamoorthy, A., & Verguts, T. (2012). Word and deed: A computational model of instruction following. Brain Research, 1439, 54–65. DOI: 10.1016/j.brainres.2011.12.025 22264490

[42] R Development Core Team. (2014). R: A language and environment for statistical computing. Vienna, Austria.

[43] Ridderinkhof, K. R., van den Wildenberg, W. P. M., Segalowitz, S. J., & Carter, C. S. (2004). Neurocognitive mechanisms of cognitive control: The role of prefrontal cortex in action selection, response inhibition, performance monitoring and reward-based learning. Brain and Cognition, 56, 129–140. DOI: 10.1016/j.bandc.2004.09.016 15518930

[44] Rouder, J. N., Morey, R. D., Speckman, P. L., & Province, J. M. (2012). Default Bayes factors for ANOVA designs. Journal of Mathematical Psychology, 56(5), 356–374. DOI: 10.1016/j.jmp.2012.08.001

[45] Rushworth, M. F. S., Passingham, R. E., & Nobre, A. C. (2005). Components of attentional set-switching. Experimental Psychology, 52, 83–98. DOI: 10.1027/1618-3169.52.2.83 15850156

[46] Schevernels, H., Bombeke, K., Van der Borght, L., Hopf, J.-M., Krebs, R. M., & Boehler, C. N. (2015). Electrophysiological evidence for the involvement of proactive and reactive control in a rewarded stop-signal task. NeuroImage, 121, 115–125. DOI: 10.1016/j.neuroimage.2015.07.023 26188262

[47] Shanks, D. R. (2010). Learning: From association to cognition. Annual Review of Psychology, 61, 273–301. DOI: 10.1146/annurev.psych.093008.100519 19575617

[48] Strayer, D. L., & Kramer, A. F. (1994). Aging and skill acquisition: Learning performance distinctions. Psychology and Aging, 9, 589–605. DOI: 10.1037/0882-7974.9.4.589 7893430

[49] Umbach, V. J., Schwager, S., Frensch, P. A., & Gaschler, R. (2012). Does explicit expectation really affect preparation? Frontiers in Psychology, 3, 1–12. DOI: 10.3389/fpsyg.2012.00378 23248606 PMC3521289

[50] Veling, H., Aarts, H., & Stroebe, W. (2012). Using stop signals to reduce impulsive choices for palatable unhealthy foods. British Journal of Health Psychology. DOI: 10.1111/j.2044-8287.2012.02092.x 23017096

[51] Veling, H. P., Aarts, H., & Papies, E. K. (2011). Using stop signals to inhibit dieters’ responses toward palatable foods. Behaviour Research and Therapy, 49, 771–780. DOI: 10.1016/j.brat.2011.08.005 21906724

[52] Verbruggen, F., Best, M., Bowditch, W. A., Stevens, T., & McLaren, I. P. L. (2014). The inhibitory control reflex. Neuropsychologia, 65, 263–278. DOI: 10.1016/j.neuropsychologia.2014.08.014 25149820

[53] Verbruggen, F., & Logan, G. D. (2008a). Response inhibition in the stop-signal paradigm. Trends in Cognitive Sciences, 12, 418–24. DOI: 10.1016/j.tics.2008.07.005 18799345 PMC2709177

[54] Verbruggen, F., & Logan, G. D. (2008b). Automatic and controlled response inhibition: Associative learning in the go/no-go and stop-signal paradigms. Journal of Experimental Psychology: General, 137, 649–72. DOI: 10.1037/a0013170 18999358 PMC2597400

[55] Verbruggen, F., & Logan, G. D. (2009a). Proactive adjustments of response strategies in the stop-signal paradigm. Journal of Experimental Psychology: Human Perception and Performance, 35, 835–54. DOI: 10.1037/a0012726 19485695 PMC2690716

[56] Verbruggen, F., & Logan, G. D. (2009b). Models of response inhibition in the stop-signal and stop-change paradigms. Neuroscience & Biobehavioral Reviews, 33(5), 647–661. DOI: 10.1016/j.neubiorev.2008.08.014 18822313 PMC2696813

[57] Verbruggen, F., & Logan, G. D. (2017). Control in Response Inhibition In: Egner, T. (Eds.), The Wiley Handbook of Cognitive Control, 97–110. Chichester, West Sussex, UK: John Wiley & Sons DOI: 10.1002/9781118920497.ch6

[58] Verbruggen, F., McAndrew, A., Weidemann, G., Stevens, T., & McLaren, I. P. (2016). Limits of executive control: Sequential effects in predictable environments. Psychological Science, 27(5), 748–757. DOI: 10.1177/0956797616631990 27000177 PMC4873728

[59] Verbruggen, F., McLaren, I. P. L., & Chambers, C. D. (2014). Banishing the control homunculi in studies of action control and behaviour change. Perspectives on Psychological Science, 9, 497–524. DOI: 10.1177/1745691614526414 25419227 PMC4232338

[60] Verbruggen, F., Stevens, T., & Chambers, C. D. (2014). Proactive and reactive stopping when distracted: An attentional account. Journal of Experimental Psychology: Human Perception and Performance. DOI: 10.1037/a0036542 PMC4120704 24842070

[61] Zandbelt, B. B., Bloemendaal, M., Neggers, S. F., Kahn, R. S., & Vink, M. (2012). Expectations and violations: Delineating the neural network of proactive inhibitory control. Human Brain Mapping. DOI: 10.1002/hbm.22047 PMC6869973 22359406

