# Supplementary material for: Instructed and Acquired Contingencies in Response-Inhibition Tasks
Source: J Cogn. 2019 Feb 4;2(1):4. doi: 10.5334/joc.53 (PMC6634444; doi:10.5334/joc.53)
Supplement: Supplementary Material. — Descriptive and inferential statistics for Experiment 1 and 2. [file joc-2-1-53-s1.pdf]

Supplementary Material

Go Stimuli

We used 50 words in this study.

Natural: pear, wasp, moth, calf, plum, crow, slug, dove, toad, swan, crab, pony, deer, worm, lamb, goat, frog, hawk, rice, lion, wolf, duck, bull, bear, tree.

Human-made: tram, coil, mast, gong, harp, wand, vase, raft, sofa, drum, fork, sock, coin, jeep, shed, pill, barn, sink, flag, pipe, bowl, belt, shoe, desk, book.

Descriptive and inferential statistics for Experiment 1 and 2

	go RT				*p*(miss)				*p*(error)			
	Distractors		*No distractors*		*Distractors*		*No distractors*		*Distractors*		*No distractors*	
	M	*SD*	*M*	*SD*	*M*	*SD*	*M*	*SD*	*M*	*SD*	*M*	*SD*
**Instructed**												
Part 1												
0%-stop	781	69	726	64	0.05	0.04	0.03	0.03	0.08	0.06	0.10	0.06
20%-stop	786	66	730	66	0.06	0.04	0.04	0.03	0.08	0.06	0.09	0.05
80%-stop	831	99	756	79	0.14	0.13	0.07	0.09	0.11	0.14	0.14	0.15
Part 2												
0%-stop	754	83	705	89	0.03	0.03	0.03	0.03	0.08	0.06	0.06	0.05
20%-stop	767	83	717	94	0.02	0.02	0.02	0.02	0.07	0.05	0.07	0.05
80%-stop	825	108	770	120	0.07	0.09	0.04	0.05	0.1	0.11	0.13	0.11
Part 3												
0%-stop	767	94	710	105	0.04	0.04	0.04	0.05	0.07	0.06	0.08	0.06
20%-stop	779	107	725	101	0.05	0.06	0.03	0.04	0.08	0.06	0.08	0.06
80%-stop	835	139	773	145	0.11	0.13	0.07	0.10	0.08	0.11	0.17	0.14
**Uninstructed**												
Part 1												
0%-stop	815	90	757	80	0.06	0.05	0.04	0.03	0.1	0.08	0.11	0.09
20%-stop	819	86	758	81	0.07	0.04	0.04	0.03	0.11	0.09	0.12	0.09
80%-stop	829	92	757	99	0.07	0.09	0.07	0.08	0.1	0.11	0.12	0.11
Part 2												
0%-stop	809	138	754	131	0.06	0.07	0.03	0.04	0.09	0.08	0.09	0.09
20%-stop	807	129	753	137	0.05	0.05	0.04	0.03	0.09	0.07	0.09	0.07
80%-stop	823	140	785	133	0.04	0.08	0.05	0.08	0.06	0.12	0.11	0.10
Part 3												
0%-stop	810	128	752	134	0.06	0.06	0.04	0.04	0.11	0.10	0.10	0.07
20%-stop	812	130	748	132	0.06	0.07	0.04	0.04	0.1	0.08	0.09	0.06
80%-stop	823	137	756	143	0.04	0.07	0.04	0.06	0.11	0.15	0.11	0.14

Table S1. Overview of the no-signal data in Experiment 1. Average go reaction time (RT), the probability of a missed go response [*p*(miss)], and the probability of an incorrect go response [*p*(error)] as a function of condition, word type, distractor type, and part. *p*(miss) is the ratio of omitted responses to the total number of no-signal trials. *p*(error) is the ratio of incorrect no-signal trials to the number of correct and incorrect no-signal trials (missed trials are excluded). *M* = mean; *SD* = standard deviation.

	p(respond)				SSD			
	Distractors		*No distractors*		*Distractors*		*No distractors*	
	M	*SD*	*M*	*SD*	*M*	*SD*	*M*	*SD*
**Instructed**								
Part 1								
20%-stop	0.48	0.08	0.41	0.06	366	130	360	114
80%-stop	0.39	0.16	0.29	0.14	369	128	357	114
Part 2								
20%-stop	0.52	0.07	0.48	0.04	373	166	333	113
80%-stop	0.38	0.16	0.39	0.13	376	166	333	112
Part 3								
20%-stop	0.50	0.06	0.51	0.04	351	174	393	111
80	0.37	0.12	0.35	0.12	354	177	393	111
**Uninstructed**								
Part 1								
20%-stop	0.46	0.08	0.43	0.09	375	145	329	85
80%-stop	0.48	0.18	0.41	0.10	377	144	330	87
Part 2								
20%-stop	0.51	0.08	0.49	0.05	366	156	286	136
80%-stop	0.44	0.11	0.46	0.14	371	153	287	137
Part 3								
20%-stop	0.52	0.08	0.49	0.04	347	161	416	131
80%-stop	0.48	0.10	0.44	0.11	349	164	417	131

Table S2. Overview of the relevant stop-signal data in Experiment 1. Probability of responding on a stop trial [*p*(respond|signal)] and average stop-signal delay (SSD) as a function of condition, word type, distractor type, and part. *M* = mean; *SD* = standard deviation.

	Df1	*Df2*	*Sum of squares effect*	*Sum of squares error*	*F*	*p*	α_{adj}	*gen.* η^2
**No-signal trials: RT**								
Condition	1	46	123926.20	7639132.60	0.75	0.392	0.005	0.012
Part	2	92	6022.55	1209571.42	0.23	0.705	0.013	0.001
Type	2	92	208930.80	260449.40	36.90	**< 0.001**	0.004	0.020
Distract	1	46	720715.70	157581.96	210.39	**< 0.001**	0.003	0.067
Condition by part	2	92	7831.28	1209571.42	0.30	0.655	0.010	0.001
Condition by type	2	92	79512.53	260449.40	14.04	**< 0.001**	0.004	0.008
Condition by distract	1	46	141.16	157581.96	0.04	0.840	0.025	< 0.001
Part by type	4	184	13498.73	225370.39	2.76	0.056	0.004	0.001
Part by distract	2	92	6570.54	147124.22	2.05	0.134	0.005	0.001
Type by distract	2	92	1324.51	80018.08	0.76	0.418	0.006	< 0.001
Condition by part by type	4	184	3870.41	225370.39	0.79	0.533	0.007	< 0.001
Condition by part by distract	2	92	654.58	147124.22	0.20	0.815	0.017	< 0.001
Condition by type by distract	2	92	1174.59	80018.08	0.68	0.512	0.006	< 0.001
Part by type by distract	4	184	3844.43	295307.76	0.60	0.588	0.008	< 0.001
Condition by part by type by distract	4	184	1284.19	295307.76	0.20	0.865	0.050	< 0.001
**No-signal trials: *p*(miss)**								
Condition	1	46	0.00	1.06	0.04	0.846	0.050	< 0.001
Part	2	92	0.07	0.55	5.45	**0.008**	0.005	0.019
Type	2	92	0.11	0.26	19.46	**< 0.001**	0.004	0.033
Distract	1	46	0.06	0.08	33.63	**< 0.001**	0.003	0.018
Condition by part	2	92	0.02	0.55	1.45	0.240	0.007	0.005
Condition by type	2	92	0.09	0.26	16.18	**< 0.001**	0.004	0.027
Condition by distract	1	46	0.00	0.08	1.48	0.229	0.006	0.001
Part by type	4	184	0.02	0.60	1.68	0.184	0.006	0.007
Part by distract	2	92	0.01	0.18	2.36	0.107	0.005	0.003
Type by distract	2	92	0.00	0.20	0.61	0.480	0.010	0.001
Condition by part by type	4	184	0.01	0.60	0.62	0.572	0.017	0.002
Condition by part by distract	2	92	0.01	0.18	1.43	0.245	0.008	0.002
Condition by type by distract	2	92	0.05	0.20	12.58	**< 0.001**	0.004	0.016
Part by type by distract	4	184	0.01	0.35	0.72	0.498	0.013	0.002
Condition by part by type by distract	4	184	0.00	0.35	0.30	0.754	0.025	0.001
**No-signal trials: *p*(error)**								
Condition	1	46	0.01	4.07	0.15	0.698	0.010	0.002
Part	2	92	0.04	0.35	5.73	0.005	0.004	0.006
Type	2	92	0.13	0.64	9.48	**0.001**	0.004	0.018
Distract	1	46	0.05	0.18	12.73	**0.001**	0.003	0.007
Condition by part	2	92	0.00	0.35	0.23	0.792	0.025	< 0.001
Condition by type	2	92	0.09	0.64	6.75	0.006	0.005	0.013
Condition by distract	1	46	0.00	0.18	1.15	0.289	0.006	0.001
Part by type	4	184	0.00	0.55	0.31	0.819	0.050	0.001
Part by distract	2	92	0.00	0.34	0.27	0.733	0.017	< 0.001
Type by distract	2	92	0.06	0.29	8.81	**0.002**	0.004	0.008
Condition by part by type	4	184	0.01	0.55	0.47	0.703	0.013	0.001
Condition by part by distract	2	92	0.02	0.34	3.03	0.061	0.005	0.003
Condition by type by distract	2	92	0.01	0.29	1.20	0.293	0.007	0.001
Part by type by distract	4	184	0.01	0.63	0.99	0.388	0.008	0.002
Condition by part by type by distract	4	184	0.02	0.63	1.26	0.289	0.006	0.002

Table S3. Overview of repeated measures Analyses of Variance on the no-signal data in Experiment 1. Condition is included as a between-subjects factor, all other factors are within-subjects. In the no-signal RT analysis, incorrect and missed no-signal trials were removed. type = word type. α_{ad} = alpha-level following the sequential Bonferroni procedure to control for multiplicity. *p*s < α_{adj} are highlighted in bold.

Omitted factor(s)	Bayes Factor	Confidence interval
**Main analysis**		
Distract	0.00	±126.46%
Type	0.00	±126.61%
Condition:type	0.00	±100.21%
Condition	0.04	±103.06%
Distract by part	0.23	±100.01%
Condition by distract by part by type	0.44	±99.77%
Part	0.53	±104.66%
Condition by distract by part	0.61	±103.96%
Condition by distract by type	0.66	±112.99%
Part by type	0.92	±101.07%
Distract by type	1.26	±117.18%
Condition by distract	1.31	±131.65%
Condition by part	2.10	±127.69%
Distract by part by type	5.45	±100.85%

Table S4. No-signal RT Bayesian analysis in Experiment 1. Bayes factors < 1 indicate that the removal of the factor or interaction had a deleterious effect on the model, whereas Bayes factors > 1 indicate that the factor or interaction could be removed without impairing the fit much. Note that ‘participant’ was included as a factor for all models, but this factor is not added to the model descriptions in the tables to reduce the amount of text. distract = distractor type (no-distractor, distractor); type = word type.

	Df1	*Df2*	*Sum of squares effect*	*Sum of squares error*	*F*	*p*	α_{adj}	*gen.* η^2
**Stop-signal trials:** ***p*****(respond|signal)**								
Condition	1	46	0.28	1.52	8.48	0.006	0.005	0.044
Part	2	92	0.22	1.04	9.63	**< 0.001**	0.004	0.034
Type	1	46	0.86	1.01	39.23	**< 0.001**	0.003	0.123
Distract	1	46	0.13	0.39	14.87	**< 0.001**	0.004	0.020
Condition by part	2	92	0.01	1.04	0.52	0.562	0.008	0.002
Condition by type	1	46	0.29	1.01	13.27	**0.001**	0.004	0.045
Condition by distract	1	46	0.00	0.39	0.13	0.717	0.017	< 0.001
Part by type	2	92	0.04	0.62	3.05	0.052	0.006	0.007
Part by distract	2	92	0.09	0.62	6.61	**0.002**	0.005	0.014
Type by distract	1	46	0.00	0.28	0.29	0.591	0.013	< 0.001
Condition by part by type	2	92	0.01	0.62	0.54	0.586	0.010	0.001
Condition by part by distract	2	92	0.01	0.62	1.03	0.363	0.007	0.002
Condition by type by distract	1	46	0.00	0.28	0.00	0.975	0.050	< 0.001
Part by type by distract	2	92	0.04	0.65	2.96	0.057	0.006	0.007
Condition by part by type by distract	2	92	0.00	0.65	0.28	0.759	0.025	0.001

Table S5. Overview of repeated measures Analyses of Variance on the stop-signal data in Experiment 1. Condition is included as a between-subjects factor, all other factors are within-subjects. type = word type. α_{ad} = alpha-level following the sequential Bonferroni procedure to control for multiplicity. *p*s < α_{adj} are highlighted in bold.

	go RT				p(miss)				p(error)			
	Distractors		*No distractors*		*Distractors*		*No distractors*		*Distractors*		*No distractors*	
	M	*SD*	*M*	*SD*	*M*	*SD*	*M*	*SD*	*M*	*SD*	*M*	*SD*
**Instructed**												
Part 1												
0%-stop	913	161	880	158	0.02	0.03	0.02	0.03	0.07	0.07	0.07	0.06
20%-stop	926	159	885	149	0.02	0.03	0.02	0.03	0.07	0.05	0.08	0.06
80%-stop	969	194	927	145	0.05	0.10	0.04	0.10	0.10	0.13	0.09	0.11
Part 2												
0%-stop	968	259	922	237	0.02	0.03	0.02	0.04	0.07	0.06	0.05	0.07
20%-stop	982	264	936	238	0.02	0.03	0.02	0.03	0.06	0.05	0.06	0.06
80%-stop	1041	280	1027	262	0.03	0.08	0.03	0.08	0.08	0.09	0.06	0.09
Part 3												
0%-stop	1016	276	965	263	0.02	0.03	0.02	0.03	0.07	0.06	0.07	0.08
20%-stop	1018	276	985	264	0.02	0.02	0.02	0.02	0.07	0.06	0.07	0.07
80%-stop	1102	316	1075	311	0.02	0.04	0.03	0.06	0.09	0.10	0.15	0.16
**Uninstructed**												
Part 1												
0%-stop	927	164	870	160	0.01	0.02	0.01	0.02	0.05	0.04	0.06	0.05
20%-stop	943	164	879	156	0.02	0.02	0.01	0.02	0.05	0.04	0.06	0.03
80%-stop	953	162	904	166	0.01	0.03	0.02	0.05	0.07	0.08	0.05	0.08
Part 2												
0%-stop	1018	292	982	283	0.02	0.03	0.01	0.02	0.05	0.05	0.04	0.04
20%-stop	1032	303	974	275	0.02	0.03	0.02	0.03	0.04	0.04	0.04	0.04
80%-stop	1054	320	1010	303	0.03	0.09	0.02	0.06	0.04	0.09	0.07	0.09
Part 3												
0%-stop	1056	307	1010	297	0.02	0.04	0.02	0.04	0.05	0.05	0.05	0.06
20%-stop	1058	311	1003	289	0.03	0.04	0.02	0.03	0.05	0.05	0.05	0.06
80%-stop	1075	308	1035	293	0.03	0.05	0.02	0.05	0.06	0.11	0.06	0.10

Table S6. Overview of the no-signal data in Experiment 2. Average go reaction time (RT), the probability of a missed go response [*p*(miss)], and the probability of an incorrect go response [*p*(error)] as a function of condition, word type, distractor type, and part. *p*(miss) is the ratio of omitted responses to the total number of no-signal trials. *p*(error) is the ratio of incorrect no-signal trials to the number of correct and incorrect no-signal trials (missed trials are excluded). *M* = mean; *SD* = standard deviation.

	p(respond|signal)				SSD			
	Distractors		*No distractors*		*Distractors*		*No distractors*	
	M	*SD*	*M*	*SD*	*M*	*SD*	*M*	*SD*
**Instructed**								
Part 1								
20%-stop	0.46	0.13	0.39	0.13	465	173	587	214
80%-stop	0.35	0.17	0.33	0.17	465	178	591	215
Part 2								
20%-stop	0.48	0.12	0.47	0.06	513	205	619	221
80%-stop	0.35	0.16	0.34	0.12	519	210	623	222
Part 3								
20%-stop	0.50	0.08	0.49	0.06	493	258	465	162
80%-stop	0.36	0.17	0.36	0.18	495	261	467	167
**Uninstructed**								
Part 1								
20%-stop	0.39	0.09	0.35	0.09	565	224	613	300
80%-stop	0.41	0.12	0.32	0.10	570	227	615	300
Part 2								
20%-stop	0.48	0.06	0.47	0.06	442	166	622	307
80%-stop	0.43	0.11	0.44	0.12	446	169	625	311
Part 3								
20%-stop	0.50	0.08	0.49	0.07	415	204	553	175
80%-stop	0.44	0.14	0.46	0.13	414	204	556	178

Table S7. Overview of the relevant stop-signal data in Experiment 2. Probability of responding on a stop trial [*p*(respond|signal)] and average SSD as a function of condition, word type, distractor type, and part. *M* = mean; *SD* = standard deviation.

	Df1	*Df2*	*Sum of squares effect*	*Sum of squares error*	*F*	*p*	α_{adj}	*gen.* η^2
**No-signal trials: RT**								
Condition	1	70	60763.15	65147721.30	0.07	0.799	0.025	0.001
Part	2	140	3157371.00	10406859.00	21.24	**< 0.001**	0.004	0.038
Type	2	140	730815.40	668837.20	76.49	**< 0.001**	0.003	0.009
Distract	1	70	614976.60	708947.80	60.72	**< 0.001**	0.004	0.008
Condition by part	2	140	70810.58	10406859.00	0.48	0.555	0.010	0.001
Condition by type	2	140	169491.00	668837.20	17.74	**< 0.001**	0.004	0.002
Condition by distract	1	70	13219.55	708947.80	1.31	0.257	0.006	< 0.001
Part by type	4	280	31759.51	986425.00	2.25	0.092	0.005	< 0.001
Part by distract	2	140	2934.04	514823.60	0.40	0.662	0.013	< 0.001
Type by distract	2	140	9656.24	297829.80	2.27	0.119	0.006	< 0.001
Condition by part by type	4	280	28348.02	986425.00	2.01	0.093	0.005	< 0.001
Condition by part by distract	2	140	1066.16	514823.60	0.14	0.856	0.050	< 0.001
Condition by type by distract	2	140	3690.91	297829.80	0.87	0.422	0.007	< 0.001
Part by type by distract	4	280	4795.34	703878.20	0.48	0.672	0.017	< 0.001
Condition by part by type by distract	4	280	8200.87	703878.20	0.82	0.472	0.008	< 0.001
**No-signal trials: *p*(miss)**								
Condition	1	70	0.00	1.06	0.12	0.008	0.008	0.001
Part	2	140	0.00	0.48	0.05	0.050	0.050	< 0.001
Type	2	140	0.02	0.34	4.01	0.003	0.003	0.007
Distract	1	70	0.00	0.11	0.59	0.007	0.007	< 0.001
Condition by part	2	140	0.01	0.48	2.05	0.004	0.004	0.005
Condition by type	2	140	0.01	0.34	1.74	0.005	0.005	0.003
Condition by distract	1	70	0.00	0.11	1.71	0.005	0.005	0.001
Part by type	4	280	0.01	0.45	0.85	0.006	0.006	0.002
Part by distract	2	140	0.00	0.10	0.05	0.025	0.025	< 0.001
Type by distract	2	140	0.00	0.13	0.13	0.010	0.010	< 0.001
Condition by part by type	4	280	0.01	0.45	1.32	0.006	0.006	0.003
Condition by part by distract	2	140	0.00	0.10	2.14	0.004	0.004	0.001
Condition by type by distract	2	140	0.00	0.13	0.04	0.017	0.017	< 0.001
Part by type by distract	4	280	0.00	0.19	0.27	0.013	0.013	< 0.001
Condition by part by type by distract	4	280	0.00	0.19	1.81	0.004	0.004	0.002
**No-signal trials: *p*(error)**								
Condition	1	70	0.18	3.28	3.94	0.005	0.005	0.025
Part	2	140	0.06	0.77	5.17	0.004	0.004	0.008
Type	2	140	0.11	0.71	10.40	0.003	0.003	0.015
Distract	1	70	0.00	0.20	1.38	0.010	0.010	0.001
Condition by part	2	140	0.01	0.77	1.07	0.013	0.013	0.002
Condition by type	2	140	0.02	0.71	2.41	0.006	0.006	0.003
Condition by distract	1	70	0.00	0.20	0.04	0.050	0.050	< 0.001
Part by type	4	280	0.02	0.85	1.71	0.008	0.008	0.003
Part by distract	2	140	0.01	0.35	2.03	0.007	0.007	0.001
Type by distract	2	140	0.00	0.33	1.00	0.017	0.017	0.001
Condition by part by type	4	280	0.02	0.85	2.05	0.006	0.006	0.004
Condition by part by distract	2	140	0.02	0.35	3.23	0.004	0.004	0.002
Condition by type by distract	2	140	0.00	0.33	0.46	0.025	0.025	< 0.001
Part by type by distract	4	280	0.02	0.60	2.90	0.004	0.004	0.003

Condition by part by type by distract	4	280	0.02	0.60	2.57	0.005	0.005	0.003

Table S8. Overview of repeated measures Analyses of Variance on the no-signal data in Experiment 2. Condition is included as a between-subjects factor, all other factors are within-subjects. In the no-signal RT analysis, incorrect and missed no-signal trials were removed. type = word type. α_{ad} = alpha-level following the sequential Bonferroni procedure to control for multiplicity. *p*s < α_{adj} are highlighted in bold.

Omitted factor(s)	Bayes Factor	Confidence interval
**Main analysis**		
Part	0.00	±119.21%
Type	0.00	±94.87%
Distract	0.00	±94.69%
Condition by type	0.03	±96.26%
Condition by part	5.03	±104.23%
Condition by distract	11.28	±90.51%
Condition	13.91	±100.7%
Condition by distract by type	101.88	±96.08%
Part by type	117.19	±82.66%
Condition by part by type	123.91	±102.94%
Distract by type	126.63	±80.76%
Condition by distract by part by type	129.53	±94.28%
Distract by part	199.86	±101.93%
Distract:part by type	613.79	±95.13%
Condition by distract by part	796.95	±115.45%

Table S9. No-signal RT Bayesian analysis in Experiment 2. Bayes factors < 1 indicate that the removal of the factor or interaction had a deleterious effect on the model, whereas Bayes factors > 1 indicate that the factor or interaction could be removed without impairing the fit much. Note that ‘participant’ was included as a factor for all models, but this factor is not added to the model descriptions in the tables to reduce the amount of text. *Note:* distract = distractor type (no-distractor, distractor); type = word type.

	Df1	*Df2*	*Sum of squares effect*	*Sum of squares error*	*F*	*p*	α_{adj}	*gen.* η^2
**Stop-signal trials: *p*(respond|signal)**								
Condition	1	70	0.12	3.56	2.30	0.134	0.007	0.010
Part	2	140	0.89	3.30	18.86	< **0.001**	0.004	0.069
Type (20%-, 80%-stop)	1	70	1.10	1.26	61.03	< **0.001**	0.003	0.084
Distract	1	70	0.09	0.59	10.31	**0.002**	0.005	0.007
Condition by part	2	140	0.17	3.30	3.63	0.036	0.006	0.014
Condition by type	1	70	0.39	1.26	21.53	< **0.001**	0.004	0.031
Condition by distract	1	70	0.00	0.59	0.00	0.968	0.050	< 0.001
Part by type	2	140	0.08	1.21	4.64	0.014	0.006	0.007
Part by distract	2	140	0.13	0.96	9.70	< **0.001**	0.004	0.011
Type by distract	1	70	0.00	0.45	0.27	0.608	0.017	< 0.001
Condition by part by type	2	140	0.00	1.21	0.17	0.828	0.025	< 0.001
Condition by part by distract	2	140	0.01	0.96	0.72	0.475	0.010	0.001
Condition by type by distract	1	70	0.01	0.45	0.85	0.361	0.008	< 0.001
Part by type by distract	2	140	0.01	0.67	0.55	0.578	0.013	< 0.001
Condition by part by type by distract	2	140	0.05	0.67	5.42	0.005	0.005	0.004

Table S10. Overview of repeated measures Analyses of Variance on the stop-signal data in Experiment 2. Condition is included as a between-subjects factor, all other factors are within-subjects. type = word type. α_{ad} = alpha-level following the sequential Bonferroni procedure to control for multiplicity. *p*s < α_{adj} are highlighted in bold.

Analyses of the Eye Data Experiment 1

In Experiment 1, an EyeLink 1000 Desktop Mount camera system (SR Research, Ottawa, Canada) was calibrated at the beginning of each block. The gaze position of the right eye was tracked throughout each block (sampling rate: 250 Hz). The EyeLink was calibrated and controlled via Psychtoolbox (Cornelissen, Peters, & Palmer, 2002). Eye movement data were subsequently exported using the Eyelink Data Viewer (SR Research, Ottawa, Canada) for each participant. We generated a file with information about all fixations and a file with trial sequence information, and integrated these files using R for further analyses.

In the analyses of the eye movement data, we excluded participants when no fixation was registered at the beginning of the trial event (i.e. the presentation of the word) on more than 15% of trials, as this could indicate that eye-movement registration was suboptimal. Based on this criterion, we excluded two participants (1 in the instructed condition, 1 in the uninstructed condition). Note that the inclusion of these participants did not substantially alter the overall pattern of behavioural results (not shown). We also excluded all fixations that were off screen (0.1%).

In the analyses of the eye data, we focused on the number of fixations and the fixation location for two intervals: (1) the interval between the word presentation and the response on no-signal trials, and (2) after the stop signal on stop-signal trials. Eye-movements made 400 ms after the presentation of the stop signal were excluded; this value is based on the cut-off previously used in Verbruggen, Stevens, et al. (2014) as the design used in the present study was suboptimal to accurately determine the SSRTs (which were used as the cut-off in Verbruggen, Stevens, et al., 2014).

The descriptive statistics are in Tables S11-S14 and the inferential statistics are in Tables S15-S16. If the number of fixations for a particular interval = 1, then the participant did not move their eyes during this time. Based on Verbruggen, Stevens, et al. (2014), we analysed the distance between the fixated location and the centre of the screen [distance = sqrt(x-coordinate^2 + y-coordinate^2)]. Tables S13 and S14 show the proportion of fixations that fell within 4 pre-defined regions: a region around the word in the centre of the screen, a region in which the distractors occurred, a region around the stop-signal, and an outside region; these regions with their coordinates are depicted in Figure S1. Consistent with Verbruggen, Stevens, et al (2014), participants did not make many fixations during the

intervals of interest, suggesting that they mostly fixated on the centre of screen (Tables S15 and S16). Furthermore, numerical differences between groups were very small.

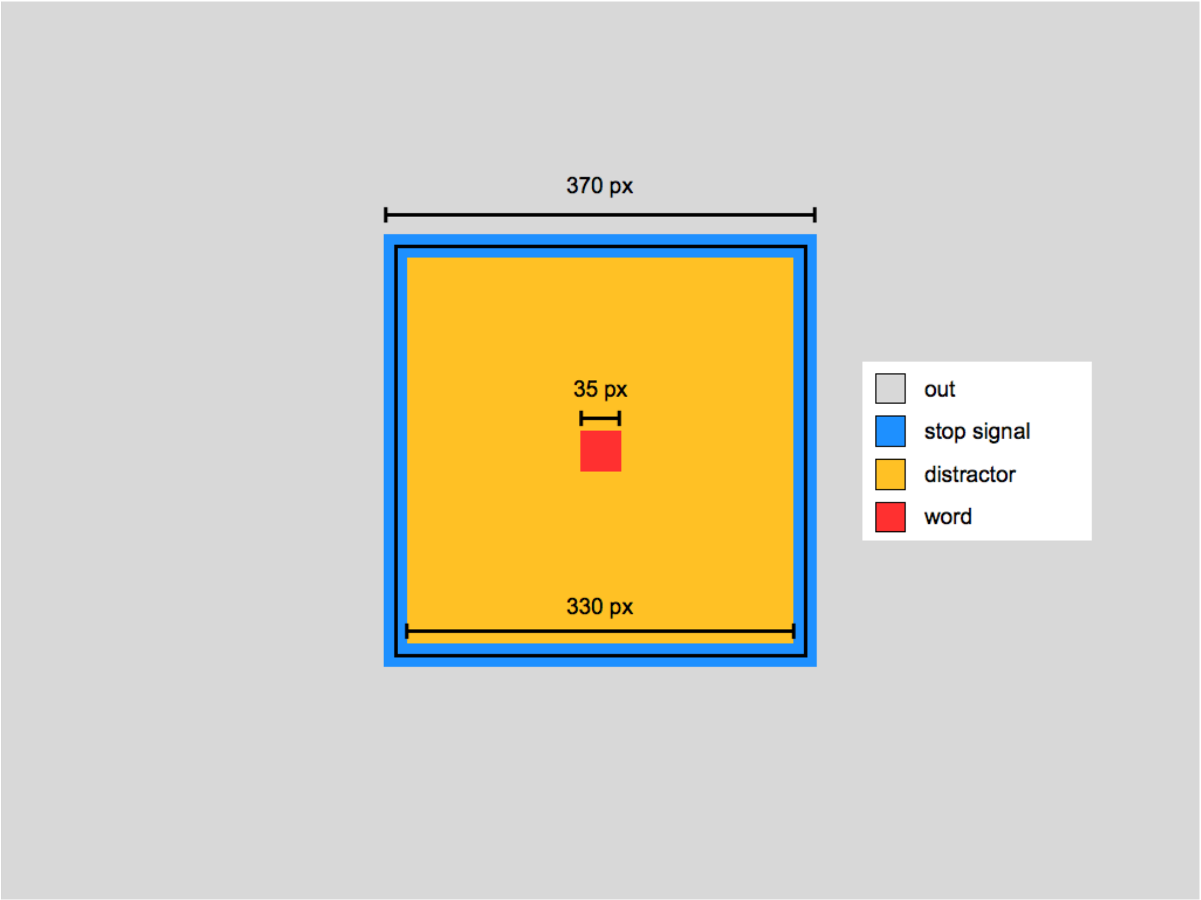

Figure S1. To analyse fixation location, we predefined 4 regions (squares): a central region around the word, a region with the distractors, a region around the stop signal, and an outside region. The size of each square is in pixels (as pixel coordinates were used for registration of fixation location). Screen size: 1024 x 768 pixels.

	Fixation				Distance			
	Distractors		*No distractors*		*Distractors*		*No distractors*	
	M	*SD*	*M*	*SD*	*M*	*SD*	*M*	*SD*
**Instructed**								
Part 1								
0%-stop	1.38	0.41	1.35	0.38	40.31	19.39	39.00	19.55
20%-stop	1.41	0.44	1.37	0.39	42.71	21.47	39.83	19.31
80%-stop	1.44	0.53	1.39	0.45	38.45	20.31	39.14	23.03
Part 2								
0%-stop	1.36	0.51	1.35	0.46	40.49	25.89	38.86	25.14
20%-stop	1.38	0.50	1.35	0.48	41.11	26.04	38.67	25.41
80%-stop	1.45	0.63	1.46	0.57	39.67	26.31	40.38	25.67
Part 3								
0%-stop	1.35	0.46	1.29	0.42	39.29	24.55	37.02	23.38
20%-stop	1.35	0.48	1.31	0.45	39.55	24.38	38.69	23.22
80%-stop	1.42	0.54	1.36	0.58	38.83	24.77	38.03	24.76
**Uninstructed**								
Part 1								
0%-stop	1.26	0.31	1.27	0.29	36.74	21.62	36.92	20.94
20%-stop	1.25	0.30	1.28	0.32	36.55	20.38	37.15	20.13
80%-stop	1.29	0.36	1.33	0.39	37.44	22.25	36.05	21.61
Part 2								
0%-stop	1.20	0.27	1.22	0.26	28.66	16.47	28.44	16.71
20%-stop	1.18	0.22	1.20	0.22	28.49	15.67	27.79	15.59
80%-stop	1.15	0.23	1.19	0.30	25.78	13.83	29.01	19.04
Part 3								
0%-stop	1.13	0.14	1.18	0.20	28.86	11.71	29.30	13.63
20%-stop	1.14	0.16	1.17	0.19	29.36	12.90	29.40	13.75
80%-stop	1.11	0.17	1.19	0.29	31.48	16.36	29.59	14.18

Table S11. Overview of the number of fixations and average distance between the fixation location and the centre of the screen (in pixels) in the interval between the word presentation and the response for each condition, part, word type, and distractor type. *M* = mean; *SD* = standard deviation.

	Fixation				Distance			
	Distractors		*No distractors*		*Distractors*		*No distractors*	
	M	*SD*	*M*	*SD*	*M*	*SD*	*M*	*SD*
**Instructed**								
Part 1								
20%-stop	1.16	0.20	1.13	0.15	42.04	27.21	38.32	26.60
80%-stop	1.18	0.22	1.14	0.17	41.53	25.39	38.25	23.62
Part 2								
20%-stop	1.13	0.15	1.11	0.14	45.71	38.34	41.96	37.26
80%-stop	1.14	0.17	1.13	0.17	44.63	39.31	42.32	37.22
Part 3								
20%-stop	1.12	0.18	1.11	0.18	43.01	36.76	42.19	35.62
80%-stop	1.14	0.20	1.11	0.12	45.34	36.20	39.89	34.54
**Uninstructed**								
Part 1								
20%-stop	1.09	0.15	1.11	0.14	35.38	24.92	35.36	24.07
80%-stop	1.11	0.16	1.13	0.15	37.35	24.99	36.62	24.43
Part 2								
20%-stop	1.06	0.12	1.05	0.06	29.67	18.58	27.29	15.93
80%-stop	1.07	0.11	1.07	0.09	27.72	15.83	29.43	18.33
Part 3								
20%-stop	1.05	0.07	1.06	0.08	29.10	14.20	29.56	14.51
80%-stop	1.04	0.06	1.05	0.08	28.35	11.75	29.37	13.77

Table S12. Overview of the number of fixations and average distance between the fixation location and the centre of the screen (in pixels) in the interval after the stop signal for each condition, part, word type, and distractor type. *M* = mean; *SD* = standard deviation.

	Word region				Distractor region				Stop-signal region				Outside region			
	D		*ND*		*D*		*ND*		*D*		*ND*		*D*		*ND*	
	M	*SD*	*M*	*SD*	*M*	*SD*	*M*	*SD*	*M*	*SD*	*M*	*SD*	*M*	*SD*	*M*	*SD*
**Instructed**																
Part 1																
0%-stop	0.72	0.21	0.73	0.21	0.24	0.20	0.24	0.20	0.03	0.04	0.02	0.03	0.02	0.04	0.01	0.03
20%-stop	0.71	0.21	0.73	0.20	0.24	0.20	0.23	0.19	0.03	0.04	0.02	0.03	0.02	0.05	0.01	0.03
80%-stop	0.74	0.23	0.72	0.25	0.23	0.22	0.23	0.24	0.02	0.05	0.03	0.06	0.01	0.03	0.02	0.05
Part 2																
0%-stop	0.75	0.20	0.77	0.19	0.20	0.15	0.18	0.13	0.03	0.06	0.03	0.07	0.02	0.05	0.01	0.03
20%-stop	0.74	0.19	0.77	0.19	0.20	0.14	0.19	0.14	0.04	0.07	0.03	0.06	0.02	0.04	0.02	0.04
80%-stop	0.77	0.20	0.75	0.21	0.18	0.16	0.19	0.17	0.02	0.05	0.03	0.08	0.03	0.07	0.02	0.05
Part 3																
0%-stop	0.74	0.19	0.76	0.18	0.21	0.15	0.20	0.14	0.03	0.06	0.02	0.06	0.02	0.05	0.01	0.04
20%-stop	0.75	0.18	0.75	0.18	0.21	0.14	0.20	0.14	0.03	0.06	0.02	0.05	0.01	0.04	0.02	0.04
80%-stop	0.78	0.19	0.77	0.21	0.17	0.14	0.19	0.17	0.04	0.06	0.02	0.04	0.01	0.04	0.02	0.03
**Uninstructed**																
Part 1																
0%-stop	0.72	0.20	0.74	0.19	0.24	0.15	0.23	0.15	0.01	0.04	0.02	0.04	0.02	0.07	0.01	0.05
20%-stop	0.73	0.17	0.73	0.19	0.23	0.14	0.24	0.15	0.01	0.03	0.02	0.03	0.02	0.07	0.02	0.06
80%-stop	0.71	0.21	0.74	0.23	0.26	0.18	0.23	0.19	0.01	0.04	0.02	0.04	0.02	0.07	0.02	0.07
Part 2																
0%-stop	0.83	0.17	0.82	0.18	0.15	0.14	0.16	0.16	0.01	0.03	0.01	0.03	0.01	0.04	0.01	0.03
20%-stop	0.84	0.16	0.84	0.16	0.14	0.14	0.14	0.14	0.01	0.02	0.01	0.02	0.01	0.04	0.01	0.03
80%-stop	0.87	0.17	0.84	0.19	0.12	0.16	0.13	0.15	0.00	0.02	0.01	0.04	0.01	0.03	0.01	0.05
Part 3																
0%-stop	0.79	0.20	0.79	0.20	0.20	0.20	0.21	0.19	0.01	0.02	0.00	0.01	0.00	0.01	0.00	0.01
20%-stop	0.79	0.20	0.79	0.20	0.20	0.19	0.20	0.19	0.00	0.01	0.01	0.02	0.01	0.02	0.00	0.01
80%-stop	0.78	0.21	0.81	0.18	0.20	0.20	0.17	0.16	0.01	0.03	0.01	0.04	0.01	0.04	0.01	0.02

Table S13. Overview of the proportion of the fixations that fell within the 4 pre-defined regions during the interval between the word and the response on no-signal trials in the interval between the word presentation and the response for each condition, part, word type, and distractor type. *D* = distractors; *ND* = No distractors; *M* = mean; *SD* = standard deviation.

	Word region				Distractor region				Stop-signal region				Outside region			
	D		*ND*		*D*		*ND*		*D*		*ND*		*D*		*ND*	
	M	***SD***	***M***	***SD***	***M***	***SD***	***M***	***SD***	***M***	***SD***	***M***	***SD***	***M***	***SD***	***M***	***SD***
**Instructed**																
Part 1																
20%-stop	0.73	0.23	0.73	0.23	0.21	0.20	0.24	0.21	0.04	0.05	0.02	0.04	0.02	0.06	0.02	0.05
80%-stop	0.71	0.24	0.72	0.25	0.24	0.21	0.24	0.22	0.03	0.06	0.02	0.05	0.02	0.05	0.01	0.04
Part 2																
20%-stop	0.72	0.26	0.74	0.25	0.20	0.15	0.20	0.16	0.06	0.12	0.04	0.09	0.02	0.05	0.02	0.06
80%-stop	0.72	0.26	0.74	0.26	0.21	0.15	0.20	0.17	0.05	0.09	0.04	0.08	0.03	0.06	0.02	0.06
Part 3																
20%-stop	0.73	0.25	0.72	0.26	0.21	0.17	0.23	0.19	0.03	0.07	0.03	0.08	0.03	0.08	0.02	0.05
80%-stop	0.70	0.24	0.75	0.24	0.24	0.16	0.20	0.16	0.04	0.08	0.03	0.07	0.02	0.06	0.02	0.06
**Uninstructed**																
Part 1																
20%-stop	0.76	0.20	0.74	0.22	0.20	0.17	0.22	0.17	0.01	0.02	0.01	0.03	0.03	0.10	0.02	0.09
80%-stop	0.74	0.19	0.74	0.22	0.23	0.14	0.23	0.17	0.01	0.03	0.01	0.04	0.02	0.08	0.02	0.07
Part 2																
20%-stop	0.83	0.18	0.85	0.15	0.15	0.15	0.13	0.13	0.01	0.02	0.01	0.02	0.02	0.04	0.01	0.05
80%-stop	0.84	0.16	0.82	0.19	0.14	0.14	0.16	0.16	0.01	0.04	0.01	0.03	0.01	0.02	0.01	0.03
Part 3																
20%-stop	0.79	0.22	0.79	0.21	0.20	0.20	0.21	0.20	0.01	0.03	0.00	0.01	0.00	0.01	0.00	0.02
80%-stop	0.78	0.22	0.78	0.20	0.21	0.21	0.20	0.19	0.00	0.01	0.01	0.03	0.00	0.01	0.00	0.01

Table S14. Overview of the proportion of the fixations that fell within the 4 pre-defined regions during the interval after the stop signal in the interval after presentation of the stop signal for each condition, part, word type, and distractor type. *D* = distractors; *ND* = No distractors; *M* = mean; *SD* = standard deviation.

	Df1	*Df2*	*Sum of squares effect*	*Sum of squares error*	*F*	*p*	α_{adj}	*gen.* η^2
**Word interval**								
Number of fixations								
Condition	1	44	5.88	103.44	2.50	0.121	0.005	0.046
Part	2	88	1.00	9.13	4.82	**0.019**	0.004	0.008
Type (0%-, 20%-, 80%-stop)	2	88	0.20	1.29	6.90	**0.008**	0.003	0.002
Distract	1	44	0.00	1.99	0.00	0.965	0.050	< 0.001
Condition by part	2	88	0.36	9.13	1.74	0.191	0.006	0.003
Condition by type	2	88	0.19	1.29	6.32	**0.011**	0.004	0.002
Condition by distract	1	44	0.27	1.99	5.91	**0.019**	0.004	0.002
Part by type	4	176	0.01	1.76	0.33	0.771	0.017	< 0.001
Part by distract	2	88	0.01	1.14	0.28	0.747	0.010	< 0.001
Type by distract	2	88	0.01	1.01	0.47	0.542	0.007	< 0.001
Condition by part by type	4	176	0.14	1.76	3.43	**0.025**	0.005	0.001
Condition by part by distract	2	88	0.04	1.14	1.59	0.212	0.006	< 0.001
Condition by type by distract	2	88	0.01	1.01	0.41	0.576	0.008	< 0.001
Part by type by distract	4	176	0.00	1.62	0.11	0.925	0.025	< 0.001
Condition by part by type by distract	4	176	0.01	1.62	0.33	0.755	0.013	< 0.001
Distance								
Condition	1	44	13071.11	273765.99	2.10	0.154	0.004	0.037
Part	2	88	3448.82	43303.67	3.50	0.051	0.003	0.010
Type (0%-, 20%-, 80%-stop)	2	88	37.77	2926.34	0.57	0.536	0.010	< 0.001
Distract	1	44	70.53	3423.98	0.91	0.346	0.005	< 0.001
Condition by part	2	88	2727.41	43303.67	2.77	0.087	0.004	0.008
Condition by type	2	88	50.18	2926.34	0.75	0.450	0.007	< 0.001
Condition by distract	1	44	78.72	3423.98	1.01	0.320	0.005	< 0.001
Part by type	4	176	86.11	6146.05	0.62	0.580	0.013	< 0.001
Part by distract	2	88	18.90	3267.00	0.25	0.719	0.025	< 0.001
Type by distract	2	88	49.12	2466.27	0.88	0.381	0.006	< 0.001
Condition by part by type	4	176	83.39	6146.05	0.60	0.592	0.017	< 0.001
Condition by part by distract	2	88	11.40	3267.00	0.15	0.804	0.050	< 0.001
Condition by type by distract	2	88	54.12	2466.27	0.97	0.354	0.006	< 0.001
Part by type by distract	4	176	132.39	4606.86	1.26	0.290	0.004	< 0.001
Condition by part by type by distract	4	176	82.08	4606.86	0.78	0.495	0.008	< 0.001

Table S15. Overview of Analyses of Variance performed to compare number of fixations and the average distance in the word interval. Type = word type. α_{ad} = alpha-level following the sequential Bonferroni procedure to control for multiplicity. *p*s < α_{adj} are highlighted in bold.

	Df1	*Df2*	*Sum of squares effect*	*Sum of squares error*	*F*	*p*	α_{adj}	*gen.* η^2
**Signal interval**								
Number of fixations								
Condition	1	44	0.54	9.33	2.56	0.117	0.004	0.044
Part	2	88	0.20	1.31	6.57	0.008	0.004	0.016
Type (20%-, 80%-stop)	1	44	0.02	0.10	7.82	0.008	0.003	0.002
Distract	1	44	0.01	0.30	2.05	0.159	0.005	0.001
Condition by part	2	88	0.03	1.31	0.84	0.389	0.010	0.002
Condition by type	1	44	0.00	0.10	0.22	0.644	0.013	< 0.001
Condition by distract	1	44	0.05	0.30	7.62	0.008	0.004	0.004
Part by type	2	88	0.01	0.24	1.54	0.220	0.006	0.001
Part by distract	2	88	0.01	0.31	1.13	0.328	0.006	0.001
Type by distract	1	44	0.00	0.13	0.08	0.774	0.025	< 0.001
Condition by part by type	2	88	0.01	0.24	1.06	0.350	0.008	< 0.001
Condition by part by distract	2	88	0.01	0.31	1.09	0.340	0.007	0.001
Condition by type by distract	1	44	0.00	0.13	0.18	0.670	0.017	< 0.001
Part by type by distract	2	88	0.01	0.22	1.71	0.192	0.005	0.001
Condition by part by type by distract	2	88	0.00	0.22	0.21	0.777	0.050	< 0.001
Distance								
Condition	1	44	16193.59	338042.94	2.11	0.154	0.005	0.039
Part	2	88	564.24	46859.63	0.53	0.537	0.008	0.001
Type (20%-, 80%-stop)	1	44	1.41	686.52	0.09	0.765	0.025	< 0.001
Distract	1	44	355.66	2519.06	6.21	0.017	0.004	0.001
Condition by part	2	88	3415.95	46859.63	3.21	0.061	0.004	0.009
Condition by type	1	44	13.46	686.52	0.86	0.358	0.006	< 0.001
Condition by distract	1	44	360.40	2519.06	6.30	0.016	0.003	0.001
Part by type	2	88	21.94	1682.83	0.57	0.566	0.010	< 0.001
Part by distract	2	88	12.93	2133.38	0.27	0.762	0.017	< 0.001
Type by distract	1	44	1.37	587.69	0.10	0.750	0.013	< 0.001
Condition by part by type	2	88	33.15	1682.83	0.87	0.424	0.007	< 0.001
Condition by part by distract	2	88	8.22	2133.38	0.17	0.840	0.050	< 0.001
Condition by type by distract	1	44	42.92	587.69	3.21	0.080	0.005	< 0.001
Part by type by distract	2	88	134.67	1509.96	3.92	0.030	0.004	< 0.001
Condition by part by type by distract	2	88	58.77	1509.96	1.71	0.192	0.006	< 0.001

Table S16. Overview of Analyses of Variance performed to compare number of fixations and the average distance in the stop-signal interval. Type = word type. α_{ad} = alpha-level following the sequential Bonferroni procedure to control for multiplicity. *p*s < α_{adj} are highlighted in bold.

Pilot Experiment

We also ran a pilot version of the stop distract where no stimulus-stop information was provided (i.e. this experiment was a procedural replication of the uninstructed condition).

Method

Participants. Twenty-four students from University of Exeter participated for monetary compensation (£5) or course credit. We excluded one participant because the percentage correct no-signal trials was below 70%. The target sample size and exclusion criteria were decided in advance of data collection.

Apparatus and stimuli, procedure and analyses. The apparatus, stimuli, and procedure were identical to those of the main study, except for the following changes: the experiment was run on a 21-in iMac, we did not track eye gaze position, and we did not obtain expectancy ratings following task completion. No participants were provided with stimulus-stop contingency information. Note that unlike in the experiments reported in the main text, we did not assess stimulus-stop expectancies following task completion in the Pilot Experiment. Thus, we could not correlate expectancies with task performance.

Results and Discussion

The descriptive statistics are presented in Table S17 and the inferential statistics in Tables S18–19. As can be seen in the tables, we did not observe an interaction between word type and distractor type, replicating the findings of the uninstructed (and instructed) condition of the main study.

	No-signal RT				p(miss)				p(error)			
	Distractors		No distractors		Distractors		No distractors		Distractors		No distractors	
	M	*SD*	*M*	*SD*	*M*	*SD*	*M*	*SD*	*M*	*SD*	*M*	*SD*
**Part 1**												
0%-stop	764	102	730	96	0.03	0.03	0.04	0.04	0.11	0.07	0.10	0.06
20%-stop	766	100	738	95	0.05	0.04	0.04	0.04	0.10	0.06	0.10	0.05
80%-stop	774	121	741	109	0.04	0.07	0.02	0.05	0.11	0.11	0.14	0.12
**Part 2**												
0%-stop	788	135	762	125	0.04	0.04	0.02	0.03	0.07	0.07	0.07	0.06
20%-stop	795	140	762	127	0.04	0.03	0.03	0.03	0.07	0.06	0.07	0.04
80%-stop	800	136	765	135	0.03	0.04	0.04	0.08	0.09	0.12	0.08	0.10
**Part 3**												
0%-stop	815	145	784	131	0.05	0.04	0.03	0.03	0.07	0.07	0.07	0.06
20%-stop	811	144	785	137	0.04	0.04	0.03	0.03	0.07	0.07	0.08	0.06
80%-stop	825	148	805	162	0.03	0.06	0.03	0.06	0.08	0.11	0.08	0.14

Table S17. Overview of the go data. Probability of an incorrect go response [*p*(error)], probability of a missed go response [*p*(miss)] and average go reaction time (RT) as a function of word type, distractor type, and part. *p*(error) is the ratio of incorrect go trials to the number of correct and incorrect go trials (missed trials are excluded). P(miss) is the ratio of omitted responses to the total number of go trials. M = mean; SD = standard deviation.

	Df1	*Df2*	*SS1*	*SS2*	*F*	*p*	*Gen. eta²*
**No-signal trials: RT**							
Type	2	44	9832	49364	4.382	**0.018**	0.002
Part	2	44	187212	466213	8.834	**< 0.001**	0.028
Distract	1	22	90251	65262	30.042	**< 0.001**	0.014
Type by part	4	88	2613	91335	0.629	0.643	0.000
Type by distract	2	44	23	64422	0.008	0.992	0.000
Part by distract	2	44	721	49297	0.322	0.726	0.000
Type by part by distract	4	88	1374	80576	0.375	0.826	0.000
**No-signal trials: *p*(error)**							
Type	2	44	0.021	0.218	2.102	0.134	0.007
Part	2	44	0.091	0.279	7.148	**0.002**	0.031
Distract	1	22	0.001	0.123	0.106	0.748	0.000
Type by part	4	88	0.004	0.445	0.184	0.946	0.001
Type by distract	2	44	0.001	0.205	0.146	0.864	0.000
Part by distract	2	44	0.001	0.162	0.178	0.837	0.000
Type by part by distract	4	88	0.009	0.280	0.744	0.565	0.003
**Stop-signal trials: *p*(respond|signal)**							
Type	1	22	0.119	0.189	13.876	**0.001**	0.046
Part	2	44	0.211	0.592	7.834	**0.001**	0.079
Distract	1	22	0.052	0.113	10.108	**0.004**	0.021
Type by part	2	44	0.018	0.323	1.234	0.301	0.007
Type by distract	1	22	0.000	0.115	0.070	0.794	0.000
Part by distract	2	44	0.055	0.343	3.508	**0.039**	0.022
Type by part by distract	2	44	0.009	0.408	0.473	0.626	0.004

Table S18. Overview of repeated measures analyses of variance. In the no-signal RT analysis, incorrect and missed no-signal trials were removed. We did not analyse *p*(miss) because values were low.

**Omitted factor(s)**	**Bayes Factor**	**Confidence interval**
Part	< 0.00	±32.35%
Distract	< 0.00	±31.28%
Type	6.84	±40.51%
Distract by type	15.66	±32.81%
Distract by part	16.84	±32.03%
Distract by part by type	28.68	±29.54%
Part by type	61.62	±33.05%

Table S19. No signal RT Bayes analysis. Bayes factors < 1 indicate that the removal of the factor or interaction had a deleterious effect on the model whereas Bayes factors > 1 indicate that the factor or interaction could be removed. Note that 'participant' was included as a factor for all models, but this factor is not added to the model descriptions in the tables to reduce the amount of text. Note: distract = distractor type (no-distractor, distractor); type = item-type.

Follow-up Experiment

After we failed to observe an increased distractor effect for the 80%-stop words in the ‘instruction’ condition of the main study, we ran a follow-up experiment to examine whether participants can make trial-by-trial attentional adjustments in the stop-distractor task. There were two block-types in this follow-up experiment: (1) *pure blocks* (these were a conceptual replication of the central signal, non-central signal, and no-signal blocks used in the Verbruggen, Stevens, & Chambers (2014) study) and (2) *mixed blocks* (in which central signal, non-central signal, and no-signal trials occurred throughout the block). In both block types, each trial began with the presentation of a cue indicating where the signal could occur. In the *pure blocks*, the cue remained the same throughout the block (i.e. the central signal cue was presented on every trial in the central signal blocks; the non-central signal cue was presented on every trial in the non-central signal blocks; the no-signal cue was presented on every trial in the no-signal blocks). In the mixed blocks, all cue types occurred.

First, we predicted that participants would proactively adjust their response settings in anticipation of the (central or non-central) signals. This should result in faster responding when no signal could occur compared with when central or non-central signals could occur. We predicted that this RT difference would be observed in pure and mixed blocks.

Second, based on the findings of Verbruggen et al. (2014), we predicted that participants would also proactively adjust their attentional settings when the non-central signal could occur compared with when no signals or central signals could occur. This should result in a larger distractor effect when participants anticipated a non-central signal than when participants anticipated no- or central-signals. The critical question was whether this enlarged distractor effect would be observed in pure blocks only or also in mixed blocks.

Method

Participants. 48 students from University of Exeter participated for monetary compensation (£5) or partial course credit. The target sample size and exclusion criteria were decided in advance of data collection. One participants was removed and replaced because their accuracy on no-signal trials was < 60%.

Apparatus and stimuli, procedure and analyses. The experiment was run using Psychtoolbox (Brainard, 1997) on a 21-in iMac (screen size: 1920 × 1080).

The go stimuli were 78 four-letter words. For each participant, we created 13 subsets of 6 words (one subset per block). On each trial, two words were presented in white lowercase font (Courier, 16-point) on a black background. One of the words referred to a natural object and the other word referred to a human-made object. One word appeared above a central white line; the other word appeared below the central line. Half of the participants responded to the location of the natural object and the other half responded to the location of the human-made object using the Up or Down arrow keys on a keyboard using their right index finger. On 50% of the trials, twenty two-letter randomly generated uppercase strings were displayed as distractors (distractor trials; Figure S2). The distractors appeared in random locations within the square. There were three cue types: central signal, non-central signal, and no-signal (Figure S2).

There were four block types: central signal blocks (a visual stop signal could occur in the centre of the screen), non-central signal blocks (a visual stop signal could appear in the periphery of the screen), no-signal blocks (no stop signals appeared), and mixed-signal blocks (central signal, non-central signal, and no-signal trials occurred in a random order). In the central signal and non-central signal blocks, a stop signal occurred on 33% of all trials; all remaining trials were no-signal (i.e. go) trials. In the mixed-signal blocks, a stop signal was presented on 22% of trials (half central-signal, half non-central signal); all remaining trials were no-signal trials. Across the whole experiment, there were 22% stop-signal trials.

The task always began with a practice mixed block of 54 trials. Following the practice block, there were 12 blocks of 54 trials comprising 2 central-signal blocks, 2 non-central signal blocks, 2 no-signal blocks, and 6 mixed-signal blocks. The order of the block types was counterbalanced across participants (e.g. P1: no-signal, mixed, central signal, mixed, non-central signal, mixed …; P2: no-signal, mixed, non-central signal, mixed, central signal ...). At the beginning of each block, participants were instructed which cues would appear in the forthcoming block. At the end of each block, participants received a 15 s break and we presented as feedback to participants their mean RT on no-signal trials, the number of

no-signal errors and the number of missed no-signal responses, and the percentage of failed stops.

All trials started with the presentation of a cue which indicated where the signal was likely to appear. In central-signal, non-central, and no-signal blocks, the cue remained the same on all trials within the block (i.e. central-signal block = central-signal cue; non-central signal block = non-central signal cue; no-signal block = no-signal cue; Figure S2). In the mixed-signal blocks (54 trials per block), central-signal trials ($N = 6$) were always preceded by the central-signal cue; non-central signal trials ($N = 6$) were always preceded by the non-central signal cue; 12 no-signal trials were preceded by the central-signal cue, 12 no-signal trials were preceded by the non-central signal cue, and the remaining 18 no-signal trials were preceded by the no-signal cue.

At the beginning of each trial, the fixation line and outer square were presented. After 250 ms, the cues appeared above and below the central fixation line (Figure S2). After 750 ms, two words replaced the cues. On half of all trials in each block (distractor trials), twenty new distractors also appeared. After every 100 ms, new distractors appeared in new random locations to ensure a perceptual load throughout the whole trial. After 1500 ms, the words and distractors were replaced by a feedback message (on no-signal trials: 'correct', 'incorrect', or not quick enough' in case they did not respond before the end of the trial: on stop-signal trials: 'correct stop' or 'incorrect stop') which remained on the screen for 500 ms. The feedback was presented to encourage fast and accurate performance. The next trial started immediately after the feedback.

On central signal trials, the central line turned bold (1 to 3 pixels). On non-central signal trials, the outline of the surrounding square (350 pixels) turned block (1 to 3 pixels). The line(s) turned bold after a variable stop-signal delay (SSD). The SSD was initially set to 500 ms, and was continuously adjusted according to a one-up/one-down tracking procedure to obtain a probability of stopping of 0.50. The SSD decreased by 50 ms when the participant responded on a stop-signal trial and increased by 50 ms when the participant successfully inhibited their response. Separate tracking procedures were used for central and non-central signal trials in the central-signal, non-central signal, and mixed-signal blocks and for

distractor and no-distractor trials. The SSD values at the end of the practice block were used as SSDs at the beginning of the main task for the corresponding trial types.

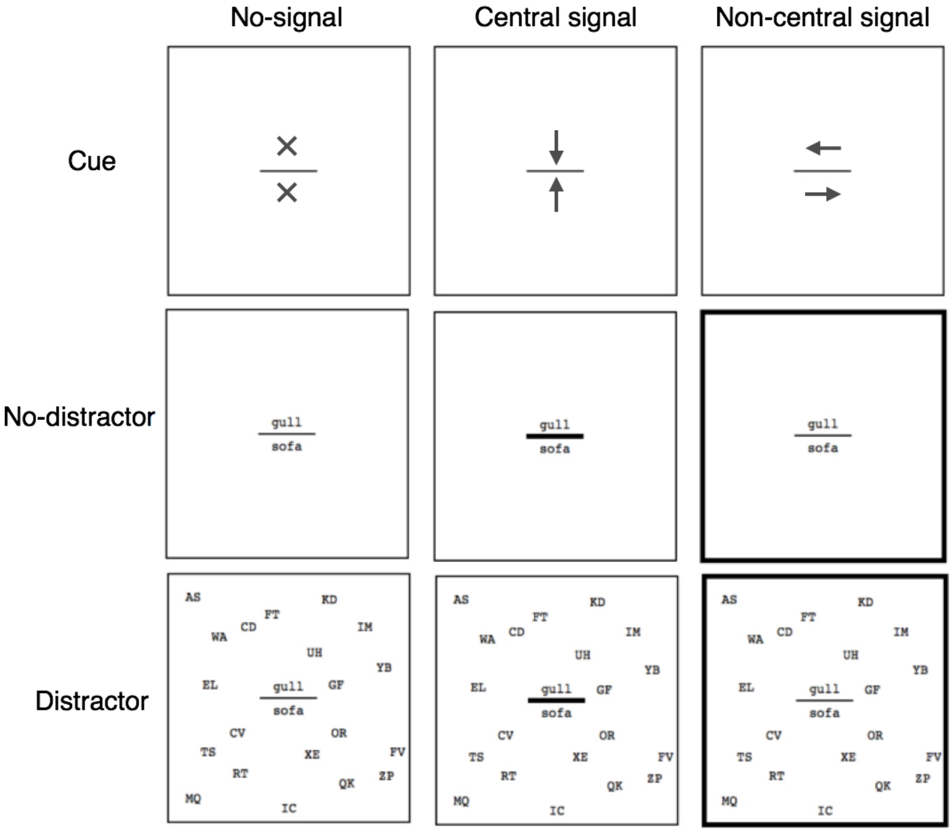

Figure S2. The distractor stop task. A cue was presented at the beginning of each trial. After 750 ms, the cue was replaced with two words presented, one above the central line and one below the central line. On half of the trials (distractor trials), random two-letter strings appeared at random locations every 100 ms. On some trials (stop-signal trials), the central fixation line turned bold (central signal) or the outer square turned bold (non-central signal) after a variable delay from the onset of the word, instructing the participants to withhold their response. For display purposes, foreground and background colours are switched in this figure. In the study, white stimuli appeared against a black background.

All data processing and analyses were completed using R (R Development Core Team, 2014). All data files and R scripts are deposited in the Open Science Framework (https://osf.io/kydw6/?view_only=131cbcc73fce4abfbfc34375819ecaad).

ANOVAs were performed on the correct no-signal RTs. We did not analyse *p*(error) or *p*(miss) because values were very low. Note also that we did not analyse performance on stop trials because we had insufficient trials to estimate SSRTs. The stop-signal delay was

continuously adjusted to obtain a probability of stopping of 0.50. Consequently, we could not use average *p*(respond|signal) as an index of inhibitory control in this study. The descriptive statistics for the go trials are presented in Table S20 and for the stop-signal trials in Table S23; frequentist statistics for no-signal RTs are presented in Table S21 and Bayesian analyses for the no-signal RTs in Table S22.

For the purpose of the analyses, the central signal blocks, non-central signal blocks, and no-signal blocks were collapsed (referred to as 'pure' blocks). Performance was analysed as a function of block-type (pure blocks, mixed blocks), cue-type (non-central, central, no-signal), distractor (distractor, no distractor). Separate ANOVAs were performed for the pure blocks and the mixed blocks. Where appropriate, we applied the Huyhn-Feldt correction for violations of sphericity. For pairwise comparisons, Hedge's g_{av} is the reported effect size measure (Lakens, 2013).

To examine support for the null hypothesis, we also computed Bayesian t-tests (Rouder, Morey, Speckman, & Province, 2012). Bayes factors compare the likelihood of the data under the null hypothesis of no difference against the alternative hypothesis of a difference with an effect size that corresponds to the prior. The default prior (0.707) assumes a medium-to-large difference between conditions. A Bayes factor less than 0.33 constitutes moderate support for the null hypothesis whereas a Bayes factor of more than 3 constitutes moderate support for the alternative hypothesis (for interpretations of Bayes factors, see Schönbrodt & Wagenmakers, 2017). We calculated the Bayes factors with the BayesFactor package in R, using the default prior of 0.707 (Morey, Rouder, & Jamil, 2015).

Based on the findings of Verbruggen et al. (2014), we had strong predictions about the direction of the RT differences between the pure central signal, pure non-central signal, and pure no-signal blocks. Furthermore, we also had strong predictions about the direction of the distractor effect following the presentation of the central signal, non-central signal, and no-signal cues. No differences or differences in the opposite direction (i.e. a smaller distractor effect following the presentation of non-central signal cues in the pure and mixed blocks) would argue against the attentional account of proactive control. Therefore, we report both the two-tailed *p*-values and the *p*-values of planned one-directional t-tests.

Results

As expected, distractors reliably slowed responding on no-signal trials by 37 ms ($p < 0.001$; Table S21). Consistent with our first prediction, there was also a reliable overall main effect of cue-type (non-central, central, no-signal; $p < 0.001$; Table S21).

The two-way interaction between block-type (pure, mixed) and cue-type was also reliable ($p < 0.001$; Table S21), indicating the cue difference was larger between pure blocks than within mixed blocks. Planned analyses in the pure signal blocks revealed that responding was faster in no-signal blocks (797 ms) than in the non-central signal blocks (991 ms), $t(47) = 10.89$, $p < 0.001$, $g_{av} = 1.84$, $BF_{10} = 3.7398e+11$ (one-tailed: $p < 0.001$), and in the central signal blocks (982 ms), $t(47) = 8.92$, $p < 0.001$, $g_{av} = 1.62$, $BF_{10} = 819729739$ (one-tailed: $p < 0.001$). There was no reliable difference between the non-central signal blocks and the central signal blocks and Bayesian analyses provided moderate support for the null hypothesis of no difference, $t(47) = -0.86$, $p = 0.395$, $g_{av} = -0.07$, $BF_{10} = 0.22$ (one-tailed: $p = 0.198$). Planned analyses in the mixed signal blocks similarly revealed that responding was faster following the presentation of the no-signal cue (848 ms) than following the presentation of the non-central signal cue (978 ms), $t(47) = 7.32$, $p < 0.001$, $g_{av} = 1.10$, $BF_{10} = 4378640$ (one-tailed: $p < 0.001$), and the central signal cue (957 ms), $t(47) = 6.01$, $p < 0.001$, $g_{av} = 0.91$, $BF_{10} = 58355.16$ (one-tailed: $p < 0.001$). Responding was also reliably slower on central signal cue trials than on non-central signal cue trials, $t(47) = 3.72$, $p = 0.001$, $g_{av} = -0.17$, $BF_{10} = 50.91$ (one-tailed: $p < 0.001$). Consistent with previous work, these results indicate that participants increased their response threshold and suppressed motor output (resulting in slower responding) when (central or non-central) signals could occur, relative to when no-signals could occur (as indicated by the cue presented at the beginning of each trial).

	RT				*p*(miss)				*p*(error)			
	Distractors		*No distractors*		*Distractors*		*No distractors*		*Distractors*		*No distractors*	
	M	*SD*	*M*	*SD*	*M*	*SD*	*M*	*SD*	*M*	*SD*	*M*	*SD*
**Pure blocks**												
Central signal cue	997	143	968	137	0.035	0.040	0.038	0.044	0.036	0.037	0.043	0.038
Non-central signal cue	1011	124	972	123	0.042	0.051	0.036	0.038	0.045	0.048	0.030	0.040
No-signal cue	817	92	777	92	0.015	0.021	0.010	0.014	0.042	0.033	0.055	0.043
**Mixed blocks**												
Central signal cue	976	125	938	128	0.036	0.043	0.030	0.036	0.047	0.047	0.043	0.036
Non-central signal cue	995	121	961	133	0.033	0.042	0.032	0.041	0.048	0.044	0.047	0.045
No-signal cue	870	114	826	112	0.024	0.031	0.020	0.023	0.045	0.035	0.041	0.034

Table S20. Overview of the no-signal data. Average no-signal reaction time (RT), probability of a missed go response [*p*(miss)], and the probability of an incorrect go response [*p*(error)] as a function of block type (pure, mixed), cue type (central, non-central signal, no-signal), and distractor type. *p*(error) is the ratio of incorrect no-signal trials to the number of correct and incorrect no-signal trials (missed trials are excluded). *p*(miss) is the ratio of omitted responses to the total number of no-signal trials. *M* = mean; *SD* = standard deviation.

Contrary to our second prediction (and the previous findings of Verbruggen, Stevens, et al., 2014), there was no reliable two-way interaction between cue-type and distractor type (p = 0.398; Table S21). The three-way interaction between block-type, cue-type, and distractor type was also not reliable (p = 0.481; Table S21). Separate analyses of the pure blocks revealed no reliable two-way interaction between cue-type and distractor type (p = 0.372; Table S21). The predicted difference between the distractor effect in non-central signal blocks and no-signal blocks also was not reliable and Bayesian analyses provided moderate support for the null hypothesis of no difference, $t(47) = 0.15$, $p = 0.879$, $g_{av} = 0.03$, BF = 0.16 (one-tailed: $p = 0.560$). Similarly, analyses of the mixed blocks revealed no reliable two-way interaction between cue-type and distractor type (p = 0.527; Table S21). The predicted difference between the magnitude of the distractor effect between the non-central signal blocks and the no-signal blocks was not reliable and Bayesian analyses provided moderate support for the null hypothesis of no difference, $t(47) = 1.01$, $p = 0.318$, $g_{av} = 0.18$, BF = 0.25 (one-tailed: $p = 0.841$). Furthermore, Bayesian ANOVAs showed that the three-way interaction between block-type, cue, and distract, and the two-way interactions between cue and distract within the pure and mixed blocks could all be dropped without impairing the model fit (Table S22).

	Df1	*Df2*	*Sum of squares effect*	*Sum of squares error*	*F*	*p*	*gen. η^2*
**Go trials: RT**							
**Pure & Mixed Blocks Combined**							
Block-type	1	47	2590.58	138047.64	0.88	0.352	< 0.001
Cue-type	2	94	3088041.00	1950806.33	74.40	**< 0.001**	0.271
Distract	1	47	196938.90	91243.80	101.44	**< 0.001**	0.023
Block-type by cue-type	2	94	162236.10	354304.00	21.52	**< 0.001**	0.019
Block-type by distract	1	47	175.05	49758.90	0.17	0.686	< 0.001
Cue-type by distract	2	94	1909.37	102780.13	0.87	0.398	< 0.001
Block-type by cue-type by distract	2	94	1283.51	82515.79	0.73	0.481	< 0.001
**Pure blocks only**							
Cue-type	2	94	2312554.00	1284223.09	84.63	**< 0.000**	0.363
Distract	1	47	92685.44	59945.22	72.67	**< 0.000**	0.022
Cue-type by distract	2	94	2141.06	103589.55	0.97	0.372	0.001
**Mixed blocks only**							
Cue-type	2	94	937723.10	1020887.24	43.17	**< 0.000**	0.181
Distract	1	47	104428.50	81057.48	60.55	**< 0.000**	0.024
Cue-type by distract	2	94	1051.82	81706.36	0.61	0.527	0.000

Table S21. Overview of the repeated measures Analyses of Variance on the no-signal data. All factors are within-subjects. *p*s < 0.05 are highlighted in bold.

Omitted factor(s)	Bayes Factor	Confidence interval
**Main analysis**		
Cue	< 0.00	±4.31%
Distract	< 0.00	±4.53%
Block-type by Cue	< 0.00	±4.80%
Block-type by Distract	7.26	±3.59%
Block-type	8.84	±4.31%
Block-type by Cue by Distract	13.11	±43.87%
Cue by Distract	23.65	±4.53%
**Pure blocks only**		
Distract	0.01	±18.66%
Cue	2.86	±18.69%
Cue by Distract	10.48	±18.69%
**Mixed blocks only**		
Cue	< 0.00	±4.93%
Distract	< 0.00	±4.43%
Cue by Distract	14.04	±4.93%

Table S22: No-signal RT Bayesian analysis. Bayes factors < 1 indicate that the removal of the factor or interaction had a deleterious effect on the model, whereas Bayes factors > 1 indicate that the factor or interaction could be removed without impairing the fit much. Note that 'participant' was included as a factor for all models, but this factor is not added to the model descriptions in the tables to reduce the amount of text. distract = distractor type (no-distractor, distractor).

	p(respond|signal)				SSD				s-r RT			
	Distractors		*No distractors*		*Distractors*		*No distractors*		*Distractors*		*No distractors*	
	M	*SD*	*M*	*SD*	*M*	*SD*	*M*	*SD*	*M*	*SD*	*M*	*SD*
**Pure blocks**												
Central signal cue	0.39	0.11	0.39	0.11	600	123	585	136	887	146	887	146
Non-central signal cue	0.41	0.13	0.41	0.11	538	168	596	113	914	147	914	147
**Mixed blocks**												
Central signal cue	0.42	0.09	0.45	0.09	550	103	531	106	847	116	806	112
Non-central signal cue	0.49	0.12	0.44	0.10	486	140	568	117	912	122	855	119

Table S23. Overview of the stop-signal data. The probability of responding on a signal trial [*p*(respond|signal)], average stop-signal delay (SSD), and signal-respond reaction time (s-r RT; the latency of incorrectly executed responses) as a function of block type (pure, mixed), cue type (central, non-central signal), and distractor type. *M* = mean; *SD* = standard deviation.

Discussion

Although we found that responding was slower on distractor trials than on no-distractor trials (which could indicate strategic adjustments of response settings or motor output), we found no reliable evidence that participants adjusted their attentional settings in the pure or mixed blocks: the magnitude of the distractor effect was similar when participants expected a non-central signal and when they expected no signal. Bayesian analyses provided moderate evidence for the null hypothesis of no difference between the non-central signal and no-signal trials in both the pure and mixed blocks. Thus, the present study provides no support for the proactive attentional adjustments reported by Verbruggen et al. (2014).

It is important to note that the presentation of a cue at the beginning of each trial could have interfered with performance in the present study. Alternatively, it is possible that the increased distractor effect in non-central signal blocks reported by Verbruggen, Stevens, et al. (2014) reflects a 'false positive'. Future research is required to distinguish between these possibilities.

References

Brainard, D. H. (1997). The psychophysics toolbox. *Spatial Vision*, *10*, 433-6.

Cai, W., Oldenkamp, C. L., & Aron, A. R. (2011). A proactive mechanism for selective suppression of response tendencies. *The Journal of Neuroscience*, 31, 5965-9. doi: 10.1523/JNEU‑ ROSCI.6292-10.2011

Cornelissen, F. W., Peters, E. M., & Palmer, J. (2002). The eyelink toolbox: Eye tracking with MATLAB and the psychophysics Toolbox. Behavior Research Methods, Instruments, & Computers, 34(4), 613–617. doi:10.3758/BF03195489

Jahfari, S., Verbruggen, F., Frank, M. J.,Waldorp, L. J., Colzato, L., Ridderinkhof, K. R., & Forstmann, B. U. (2012). How preparation changes the need for top-down control of the basal ganglia when inhibiting premature actions. *The Journal of Neuroscience*, 32, 10870-8. doi: 10.1523/JNEUROSCI.0902-12.2012

Lakens, D. (2013). Calculating and reporting effect sizes to facilitate cumulative science: A practical primer for t-tests and ANOVAs. *Frontiers in Psychology*, *4*(NOV), 1–12. doi: 10.3389/fpsyg.2013.00863

Morey, R. D., Rouder, J. N., & Jamil, T. (2015). BayesFactor: Computation of Bayes factors for common designs (Version 0.9.11-1). Retrieved from https://cran.r-project.org/web/packages/BayesFactor/

R Development Core Team. (2014). R: A language and environment for statistical computing. Vienna, Austria: R Foundation for Statistical Computing. Retrieved from http://www.R-project.org/

Rouder, J. N., Morey, R. D., Speckman, P. L., & Province, J. M. (2012). Default Bayes factors for ANOVA designs. *Journal of Mathematical Psychology*, *56*(5), 356–374. doi:10.1016/j.jmp.2012.08.001

Schönbrodt, F. D., & Wagenmakers, E. J. (2017). Bayes factor design analysis: Planning for compelling evidence. *Psychonomic Bulletin & Review*, 1-15.

Verbruggen, F., & Logan, G. D. (2009). Proactive adjustments of response strategies in the stop-signal paradigm. *Journal of Experimental Psychology: Human Perception and Performance*, 35, 835-54. doi:10.1037/a0012726

Verbruggen, F., Stevens, T., & Chambers, C. (2014). Proactive and Reactive Stopping When Distracted: An Attentional Account. *Journal of Experimental Psychology. Human Perception and Performance*, *40*(4), 1295–1300.
